# *Saccharomyces cerevisiae* fermentation product improves robustness of equine gut microbiome upon stress

**DOI:** 10.3389/fvets.2023.1134092

**Published:** 2023-02-24

**Authors:** Erika Ganda, Anirikh Chakrabarti, Maria I. Sardi, Melissa Tench, Briana K. Kozlowicz, Sharon A. Norton, Lori K. Warren, Ehsan Khafipour

**Affiliations:** ^1^Department of Animal Science, College of Agricultural Sciences, The Pennsylvania State University, University Park, PA, United States; ^2^Microbiome Center, The Pennsylvania State University, University Park, PA, United States; ^3^Cargill R&D Centre Europe, Vilvoorde, Belgium; ^4^Cargill Biotechnology R&D, Minneapolis, MN, United States; ^5^Department of Animal Sciences, University of Florida, Gainesville, FL, United States; ^6^Cargill Animal Nutrition, Minneapolis, MN, United States

**Keywords:** equine, horse, *Saccharomyces cerevisiae* fermentation product (SCFP), postbiotic, stress, microbiome

## Abstract

**Introduction:**

Nutritional and environmental stressors can disturb the gut microbiome of horses which may ultimately decrease their health and performance. We hypothesized that supplementation with a yeast-derived postbiotic (*Saccharomyces cerevisiae* fermentation product-SCFP) would benefit horses undergoing an established model of stress due to prolonged transportation.

**Methods:**

Quarter horses (*n* = 20) were blocked based on sex, age (22 ± 3 mo) and body weight (439 ± 3 kg) and randomized to receive either a basal diet of 60% hay and 40% concentrate (CON) or the basal diet supplemented with 21 g/d Diamond V TruEquine C (SCFP; Diamond V, Cedar Rapids, IA) for 60 days. On day 57, horses were tethered with their heads elevated 35cm above wither height for 12 h to induce mild upper respiratory tract inflammation. Fecal samples were collected at days 0, 28, and 56 before induction of stress, and at 0, 12, 24, and 72 h post-stress and subjected to DNA extraction and Nanopore shotgun metagenomics. Within sample (alpha) diversity was evaluated by fitting a linear model and between sample (beta) diversity was tested with permutational ANOVA.

**Results:**

The SCFP stabilized alpha diversity across all time points, whereas CON horses had more fluctuation (*P* < 0.05) at 12, 24, and 72 h post-challenge compared to d 56. A significant difference between CON and SCFP was observed at 0 and 12 h. There was no difference in beta-diversity between SCFP and CON on d 56.

**Discussion:**

Taken together, these observations led us to conclude that treatment with SCFP resulted in more robust and stable microbial profiles in horses after stress challenge.

## Introduction

The role of the microbiome and its importance has been well-established in several systems over the past two decades, including in environmental (1, 2), biomedical (3–5), and agricultural (6–8) contexts. Herbivores are particularly impacted by the gastrointestinal microbiome, given their interdependency with metabolic pathways only present in microbes that are necessary for digestion of complex carbohydrates present in forages that typify the equine daily diet.

In horses relatively fewer research studies investigating the microbiome are available, however the number of such studies is increasing rapidly (8, 9). Among the many factors that can impact the equine microbiome, stress is one of the most preeminent ones. Both diet- (10) and exercise- (11, 12) induced stress have been associated with microbiome changes in horses. Unstable microbiomes represent an open niche for opportunistic pathogen establishment and are associated with worse health outcomes. In fact, colonization resistance is one of the biggest roles played by the microbiome in maintaining host health (13–15). Thus, maintaining a robust microbiome upon stressful events would be beneficial for horse health.

Several techniques can be applied to intentionally manipulate the diversity and composition of the gut microbiome in the quest to maintain an optimal microbial community. Diet modification, pre-, pro-, and postbiotic administration, and more drastic therapeutics such as antibiotic therapy and fecal microbiota transplantation are also used for microbiome modulation (16). Postbiotics are defined as a “preparation of inanimate microorganisms and/or their components that confers a health benefit on the host” (17, 18). These preparations do not necessarily originate from probiotic microorganisms and must contain an unpurified mixture of inanimate organisms and their metabolites. Because the mode of action of postbiotics does not rely on presence of live organisms in the final product, they represent an attractive alternative for feed supplementation given their better stability during feed processing (19). Several studies evaluating the efficacy of postbiotic supplementation of *Saccharomyces cerevisiae* fermentation products (SCFP) in bovine (20, 21), avian (22), and equine (23) species have been performed. While the mechanisms by which postbiotics confer benefits to the host have not yet been completely elucidated, much of the literature indicates that postbiotic supplementation is associated with microbiome optimization (24) and improvement of immune function (20, 22, 25). However, less is known about the effects of SCFP on horses. A few recent studies have indicated improvement in immune parameters in a vaccine challenge model (23, 26) while no difference was observed in the microbiota of racehorses fed a yeast supplement (27). Taking the wealth of evidence of the beneficial effects of postbiotic administration in many species, it is reasonable to hypothesize that postbiotic administration would benefit horses under stress.

Horses are exposed to stressful situations daily, including transportation, exercise, and diet changes. Although several studies have demonstrated the impact of stressful events on the equine fecal microbiome (10, 28) little evidence is available on how postbiotic administration can impact the robustness of microbiome in horses under stress. Thus, the objective of this study was to determine if supplementation with SCFP would result in more robust microbiome in an established equine model to simulate stress due to prolonged transportation. We hypothesized that SCFP supplementation would result in a more robust microbiome that would be less impacted by experimental stress.

## Materials and methods

### Experimental design, animals, and sample collection

The animal experiment for present microbiome study was described by Tench et al. (29). The protocol for the use of experimental animals was approved by the Institutional Animal Care and Use Committee at the University of Florida in Gainesville, FL (#201810324) under the Guide for the Care and Use of Agricultural Animals in Research and Teaching (30).

**Figure 1:**
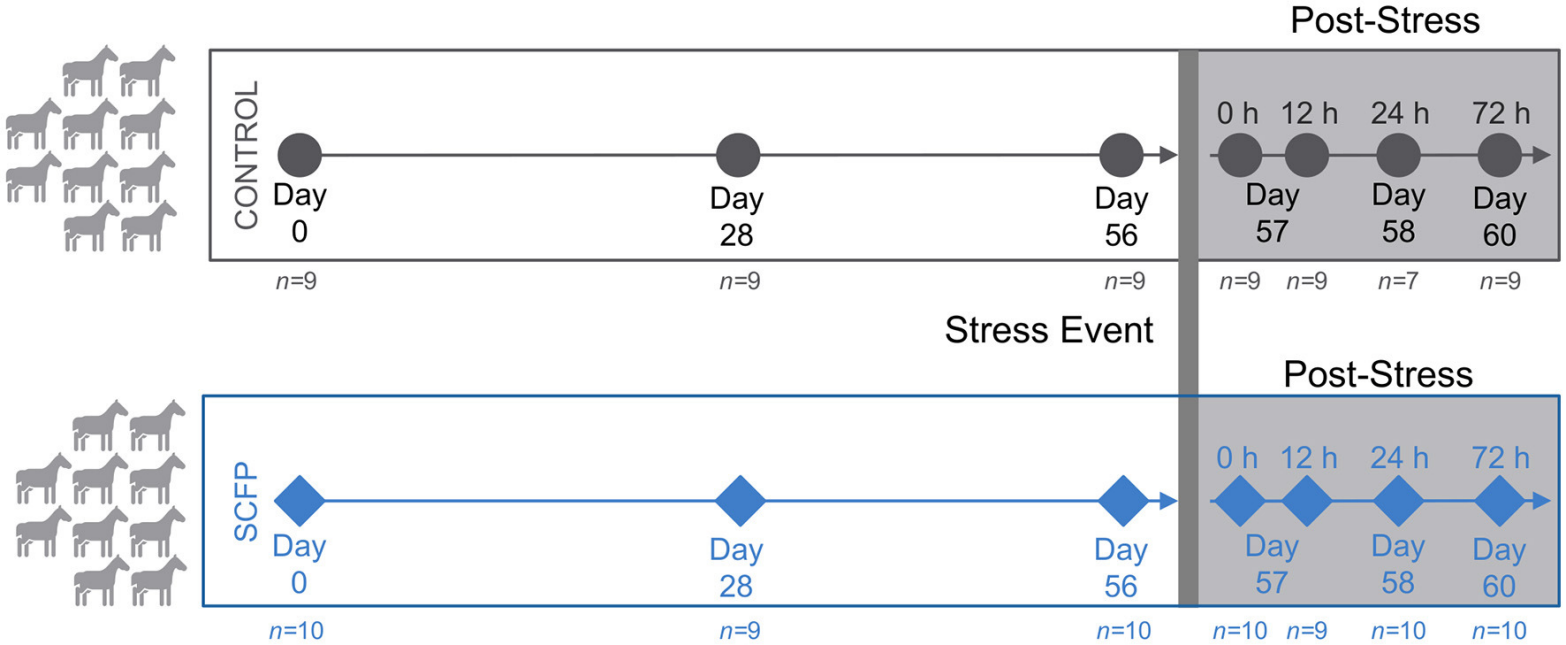
Study overview. Young and clinically healthy horses were paired by age and sex and randomly assigned into Control (*n* = 10) or *Saccharomyces cerevisiae* fermentation product (SCFP; *n* = 10). Horses received diets for 60 days. On day 57, horses were subjected to a previously established stress protocol to induce mild upper respiratory tract inflammation that mimicked long-distance transportation. Samples were collected on days 0, 28, and 56 before stress and at 0, 12, 24, and 72 h post-stress.

Briefly, 20 young and clinically healthy horses in training (mean ± SEM; initial age 22 ± 0.3 mo and BW 439 ± 3 kg) were paired by age and sex and randomly assigned to one of the two experimental treatments for 60 days. Treatments included supplementation with 0 g/d (Control; no treatment Control) or 21 g/d Diamond V TruEquine C (SCFP; Diamond V, Cedar Rapids, IA). A basal diet of 60% Coastal bermudagrass hay and 40% concentrate formulated to meet the nutrient requirements of horses at a moderate rate of growth (31) was offered to all horses. Treatment administration was done by top dressing SCFP on the concentrate ration. Horses were exercised 4 days per week for 30–45 min/d at light to moderate intensity. On day 57, horses were placed in individual stalls and tethered with their heads elevated 35 cm above wither height for 12 h to induce mild upper respiratory tract inflammation according to a previously established protocol to mimic long-distance transport stress (32, 33). Induction of inflammation was confirmed by significantly elevated serum cortisol and blood leukocyte measurements performed after stress induction compared to pre-stress (34, 35). The stress period was relieved after the 12 h timepoint by untethering of the horse heads. Fecal samples were collected into sterile containers at seven time points: days 0, 28, and 56 before induction of stress, and at 0, 12, 24, and 72 h post-stress, where 0 h is the time at which the horses were untethered. Samples were immediately placed on ice and transported to the laboratory where they were kept in a −80°C freezer until DNA extraction. A schematic of the experimental design and sample collection is given in Figure 1.

### DNA extraction and shotgun metagenomic sequencing

#### DNA extraction

Fecal samples were removed from the −80°C freezer 1 day prior to DNA extraction and thawed in a 4°C refrigerator overnight. The ZymoBIOMICS 96 MagBead DNA kit (Zymo Research Corporation, Irvine, CA) was used in a Biomek i7 (Beckman Coulter, Indianapolis, IN) workstation for DNA extraction according to manufacturer's instructions. Four extraction blanks were included in each 96 well plate to confirm that cross contamination did not occur.

#### Nanopore sequencing

Libraries were constructed using the SQK-RPB004 Rapid PCR Barcoding kit (ONT, Oxford, UK). Library preparation included DNA extraction blanks for quality control. Shotgun metagenomic sequencing was performed using R9.4.1 FLO-MIN 106 flow cells on the GridION platform (ONT, Oxford, UK), multiplexing 12 samples in each flow cell. Each sequencing run lasted 70 h. The MinKNOW ONT software (v 3.6.5) with Guppy basecaller was used for sequencing using the high-accuracy basecalling setting, followed by de-multiplexing, adapter trimming, and quality control using default settings.

### Bioinformatics and statistical analyses

#### Taxonomic assignment and microbial diversity

Fastq files obtained from the MinKNOW ONT workflow were used for microbial taxonomic classification. First, host DNA was removed by mapping fastq files to the horse genome (assembly EquCab3.0) using Minimap2 (36) followed by the removal of any reads matching the horse genome using SAMtools (37). The remaining reads were assumed to be from microbial origin and used for taxonomic assignment. To improve microbial classification, a custom database was made, which contained high quality genomes from the RefSeq database (38) and published metagenome-assembled genomes (38–40). The Kraken2 pipeline (41) was used for species identification and Bracken was used to estimate species abundances (42). Diversity metrics were calculated in R (43) using the Phyloseq package (44, 45) with the rarefied species count table from Bracken as input. Species tables were center-log transformed using the microbiome package (46) after imputation of zeros using a Bayesian multiplicative replacement method from the zCompositions package (47). Species with non-zero presence in at least 75% of samples and relative abundance >0.001% were identified separately in the pre-stress and post-stress periods and the superset containing all species was used for differential abundance analysis.

#### Functional potential

To have a better understanding of the microbiome functional potential, the Carbohydrate-Active enZymes (CAZy) (48) present in the microbiome communities for each sample were identified. First, genomes from microbial species identified with Kraken2 were annotated using PROKKA (45), followed by additional assessment of gene function using EggNOG-mapper v2 (49). After the annotation process was completed, a custom python script was used to compile the CAZy for each genome, generating a table with the accumulated CAZy potential for all the microbes identified for each sample. Results were compiled into a final table containing numbers of annotated features for each sample.

#### Statistical analyses

##### Diversity metrics

Within sample (alpha) diversity was evaluated by fitting a linear model with the lmer function of the lme4 package (50) in R. The model included Shannon diversity index as the dependent variable, horse as a random effect, treatment, timepoint, and their interactions as independent variables. Because stress is nested within timepoint, the effect of stress only is evaluated in a separate model. Between sample (beta) diversity was tested with permutational ANOVA using the adonis function in the vegan package (51) in R. The model included Aitchison distances (52) calculated based on CLR transformed values as the dependent variable, and treatment, timepoint, and their interactions as independent variables. Data was visualized with PCA using the Phyloseq package (44, 45).

##### Differential abundance

A modified version of the linear discriminant analysis from the LinDA package (53) was used to fit linear models that included relative abundances as the dependent variable, treatment, timepoint, and their interaction as independent variables, and horse as a random effect. The output from each model was then analyzed with the emmeans package (54) to calculate fold changes of centralized log ratio (CLR) transformed data of each measurement (species) for each animal with respect to their initial sample collected at day 0. False discovery rate (FDR) correction (55, 56) was used to identify species within each timepoint that significantly differed between treatment and Control.

##### Correlation networks

An adaptation of the CoNet framework (57), which includes generation of a combination of diverse measures of correlation (including Pearson's, Spearman's, and Kendall's correlation coefficients) using CLR transformed data was used for correlation network analyses. Distributions of all pair-wise scores between the nodes were computed for each timepoint. Only edges (correlations) with *p*-values < 0.05 after FDR correction (55, 56) were taken into further consideration, and edges not supported by at least two measures were discarded.

##### Clustering

Identification of the optimal number of clusters and clustering was calculated and performed using gap statistics (58) in MATLAB R2019b (59) using the spearman correlation for species and CAZy identifiers, and Aitchison distance for samples. The difference of the CLR transformed values at any time point and its corresponding value at day 0 were used as the input. The data was sorted based on experimental variables or clusters and visualized.

## Results

### Sequencing parameters

A total of 140 samples were sequenced. On average, 389,680 reads were obtained per sample (mean 389,680, median 377,834, SD 118,596). Read N50 lengths averaged 4,043 bp (mean 4,043 median 4,052, SD 318). Reads had an average quality score of 12 (mean 12, median 12, SD 0.6). On average, 1,429,212,272 total bases were obtained per sample, with a standard deviation of 413,891,226 bases per sample. Four samples had low sequencing throughput and were removed from further analysis.

### Taxonomic assignment

On average, 67% of reads were assigned at the species level (mean 66.9%, median 67.4%, SD 4.4%). A total of 119 taxa were identified (Supplementary Table 5). Of those, 27 taxa fit the criteria of being present in at least 75% of samples and relative abundance >0.001% in the pre-stress period and 18 taxa fit the criteria in the post-stress period. The final superset that was used for differential abundance analysis contained 27 taxa.

### Stress significantly impacts microbial diversity and SCFP treatment leads to a more robust microbiome after stress

**Figure 2:**
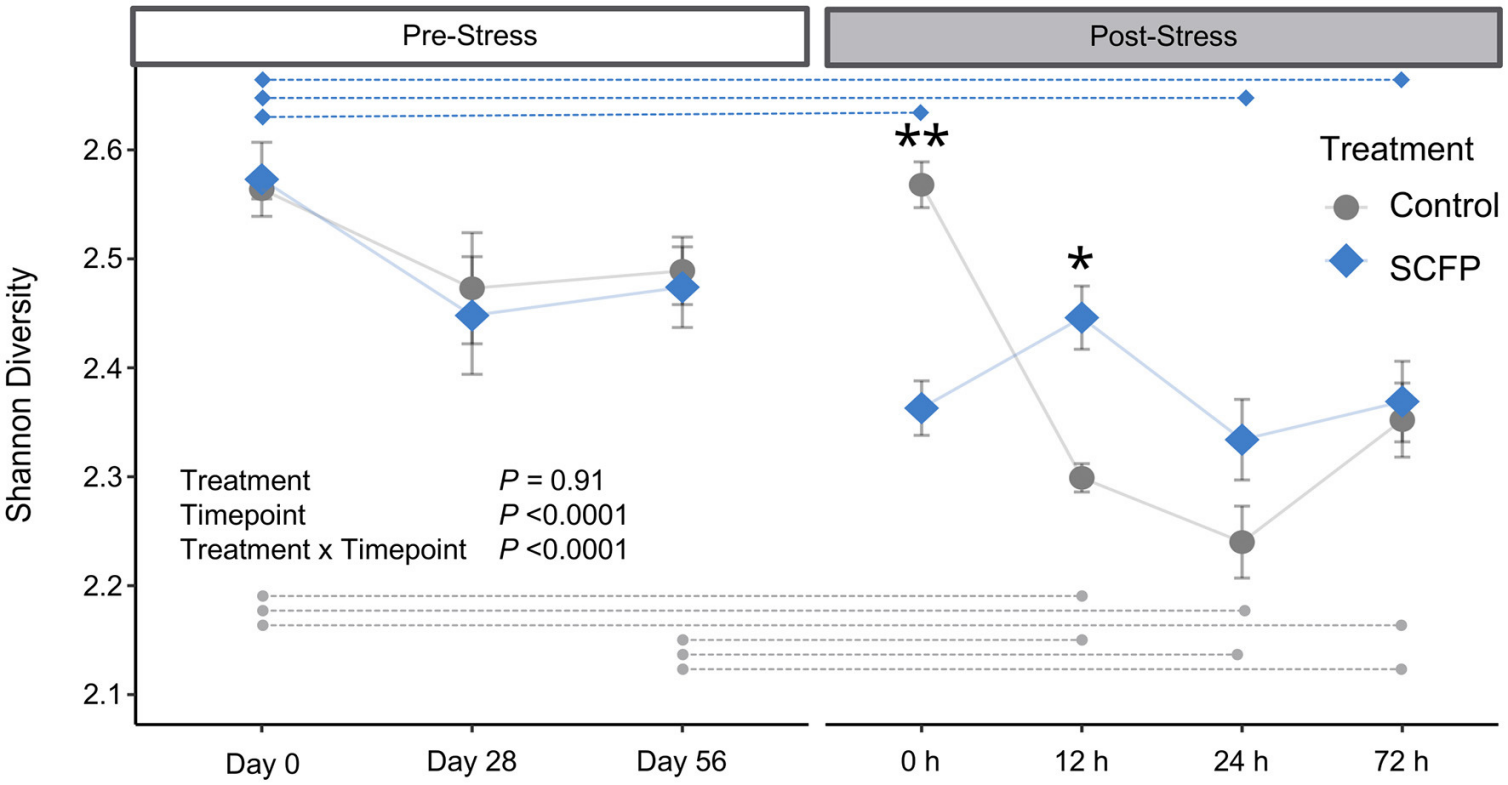
Alpha diversity comparisons. Shannon diversity metrics were analyzed with a linear model which included horse as a random effect, treatment, timepoint, and their interactions. Raw means and standard deviations are shown in the plot. Blue diamonds represent SCFP, and gray circles represent Control. Transparent lines represent the hypothetical trajectory of diversity. Dashed horizontal lines represent within-group pairwise significant differences at the 0.05 level after Bonferroni multiple comparison adjustment. Asterisks indicate timepoints in which SCFP significantly differs from Control **P < 0.01, *P < 0.05.

Alpha diversity was similar between Control and SCFP groups in the pre-stress period (Figure 2), indicating treatment with SCFP did not significantly alter Shannon microbial diversity index values. Stress impacted (*P* < 0.0001) diversity levels both in the Control and SCFP groups. However, stress had a lower impact in changing the SCFP group's diversity levels when compared to Controls. Overall, horses treated with SCFP exhibited robust microbial diversity after stress, with less variation and overall lower stress-induced drop in diversity when compared to the Control group (Supplementary Table 1). When within-group comparisons were made, statistical differences were observed in the Control group between several timepoints (Figure 2, gray dotted lines; Supplementary Table 2). On the other hand, fewer timepoints were significantly different from one another when within-group comparisons were made in the SCFP group (Figure 2, blue dotted lines; Supplementary Table 3), indicating that SCFP treatment might have contributed to more stable diversity levels post-stress.

**Figure 3:**
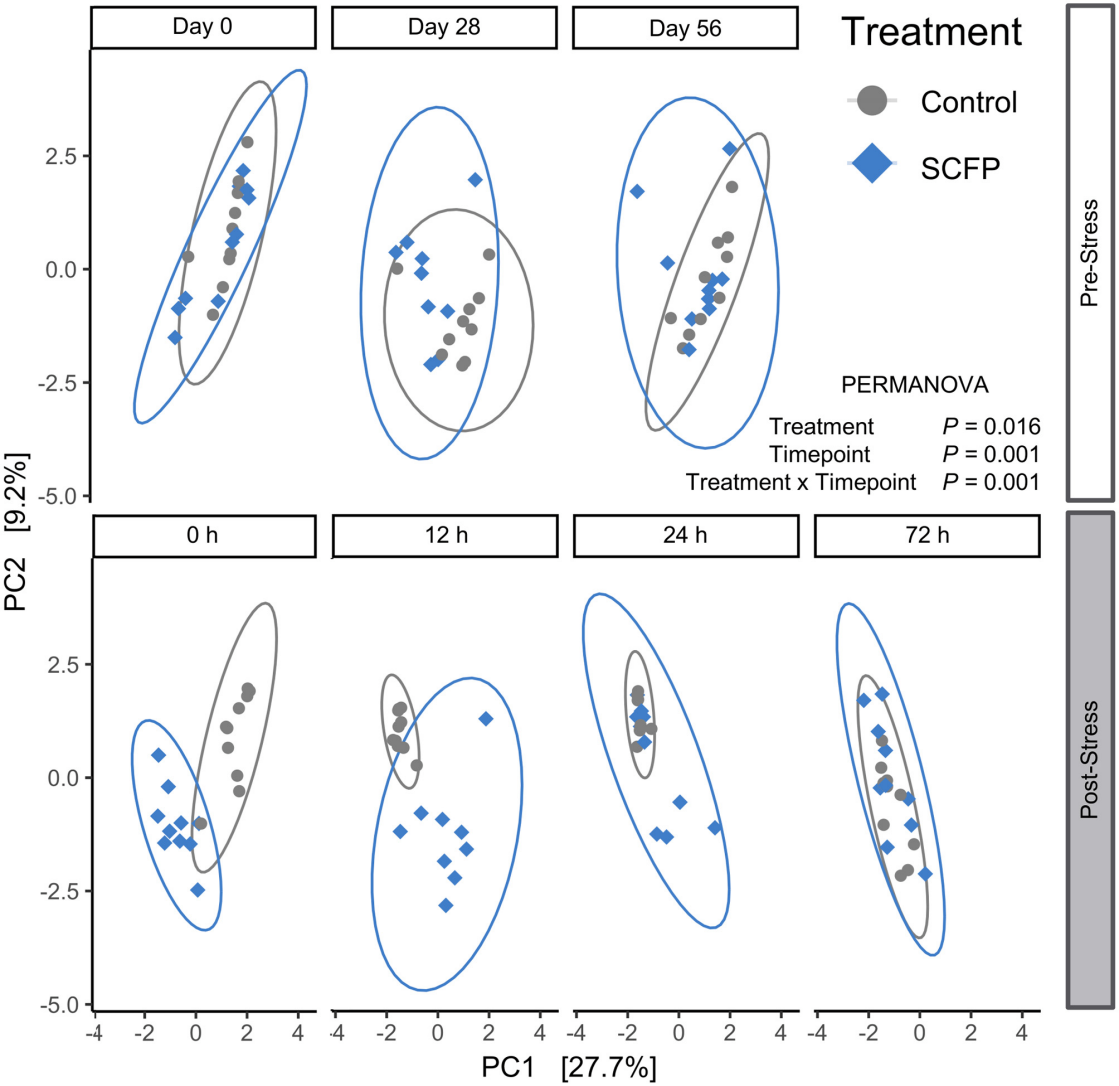
Beta diversity comparisons. Aitchison distances were analyzed with permutational ANOVA (PERMANOVA) model which included treatment, timepoint, and their interactions. Plots depict principal component analysis of Aitchison distances with CLR transformed data.

Beta diversity was variable in the pre-stress period (Figure 3). At time 0 h (time at which the horses were untethered), horses assigned to the SCFP treatment formed two subclusters, whereas horses assigned to the Control treatment clustered in the same overall region (Figure 3, panel 1). On day 28, treated and untreated horses clustered in two overlapping groups (Figure 3, panel 2), and became homogeneous over time, with no clear difference between Control and SCFP-treated horses on day 56 (Figure 3, panel 3). However, Control and SCFP-treated horses had two completely different clustering trajectories after stress, with SCFP and Control horses clustering separately at 0 and 12 h post-stress (Figure 3, panels 4 and 5) and culminating again in a homogenous group at 72 h post-stress (Figure 3, panel 7).

### The stress impact was greater for Control horses

**Figure 4:**
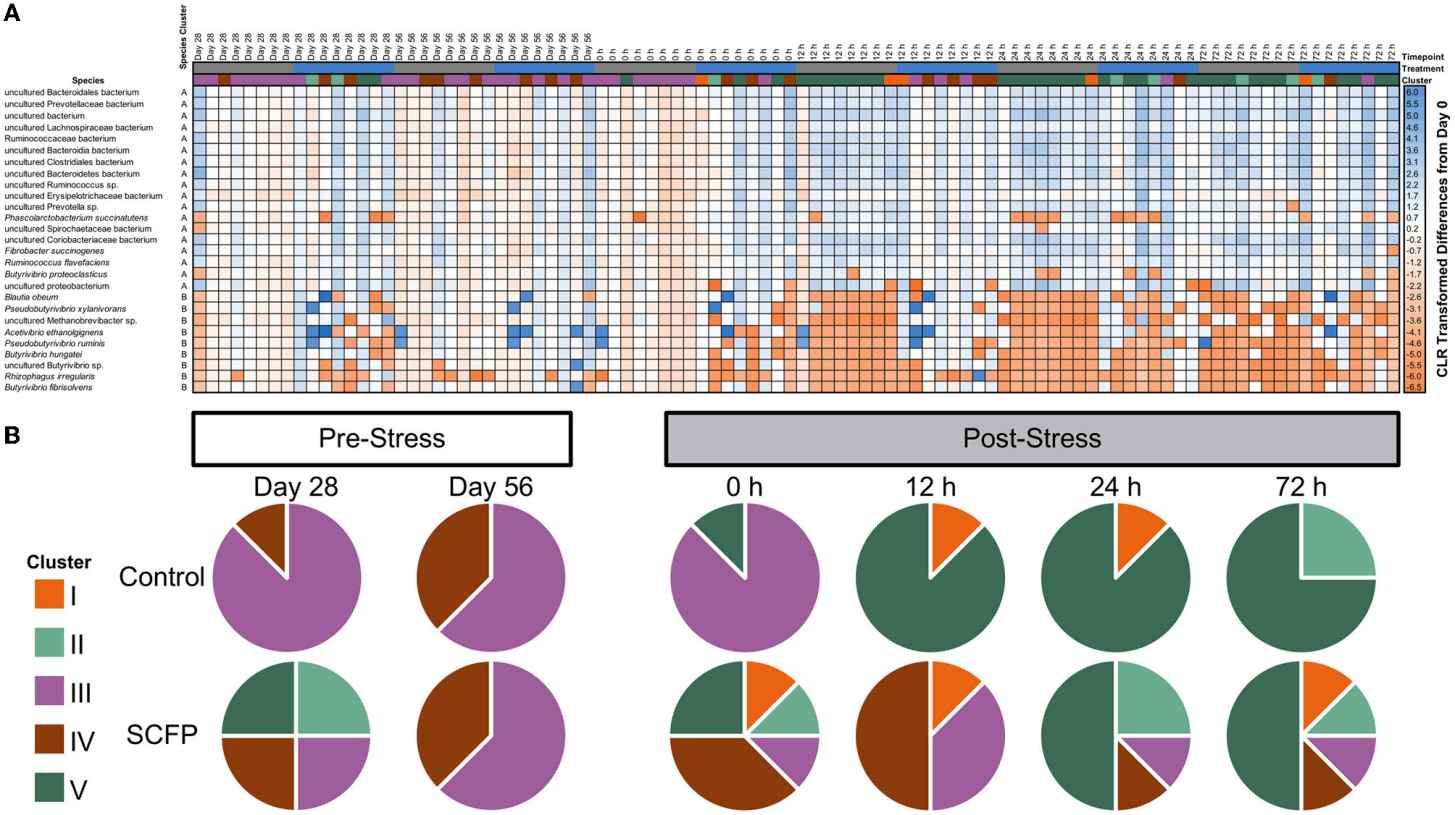
Stress challenge results in different microbial profiles in Control and SCFP treated horses. **(A)** Heatmap of centered log-ratio relative abundance of bacterial species detected in fecal samples of horses. **(B)** Pie charts represent the presence of samples across different clusters for Control and SCFP groups across time. Species cluster A contains species that drastically increase in relative abundance upon stress challenge, whereas species cluster B is comprised of species which decrease after stress challenge.

Stress challenge and SCFP treatment significantly influenced microbial composition at the species level (PERMANOVA of Aitchison distances: Treatment, *P* = 0.01; Timepoint, *P* = 0.01; Treatment × Timepoint, *P* = 0.01). Two species clusters were identified (Figure 4A, vertical clusters A and B). The larger cluster (cluster A−18 species) comprised mainly species that increased in abundance after stress challenge. The smaller cluster (cluster B—nine species) comprised species that decreased in abundance after stress challenge (Figure 4A, vertical clusters A and B, Supplementary Table 5). Notably, Control horses had a much more marked reduction in species belonging to cluster B after stress when compared to those treated with SCFP. When total microbial composition was used as a basis for clustering analysis of the samples, five major sample clusters were identified (Figure 4A, clusters I, II, III, IV, and V). Very different trajectories were observed between the SCFP and Control treatments after stress (Figure 4B), with microbial composition of Control horses mostly belonging to cluster V, while SCFP treated horses exhibited microbiome compositions representatives of all clusters.

### Stress challenge resulted in significant differential abundances in a time-dependent manner

**Figure 5:**
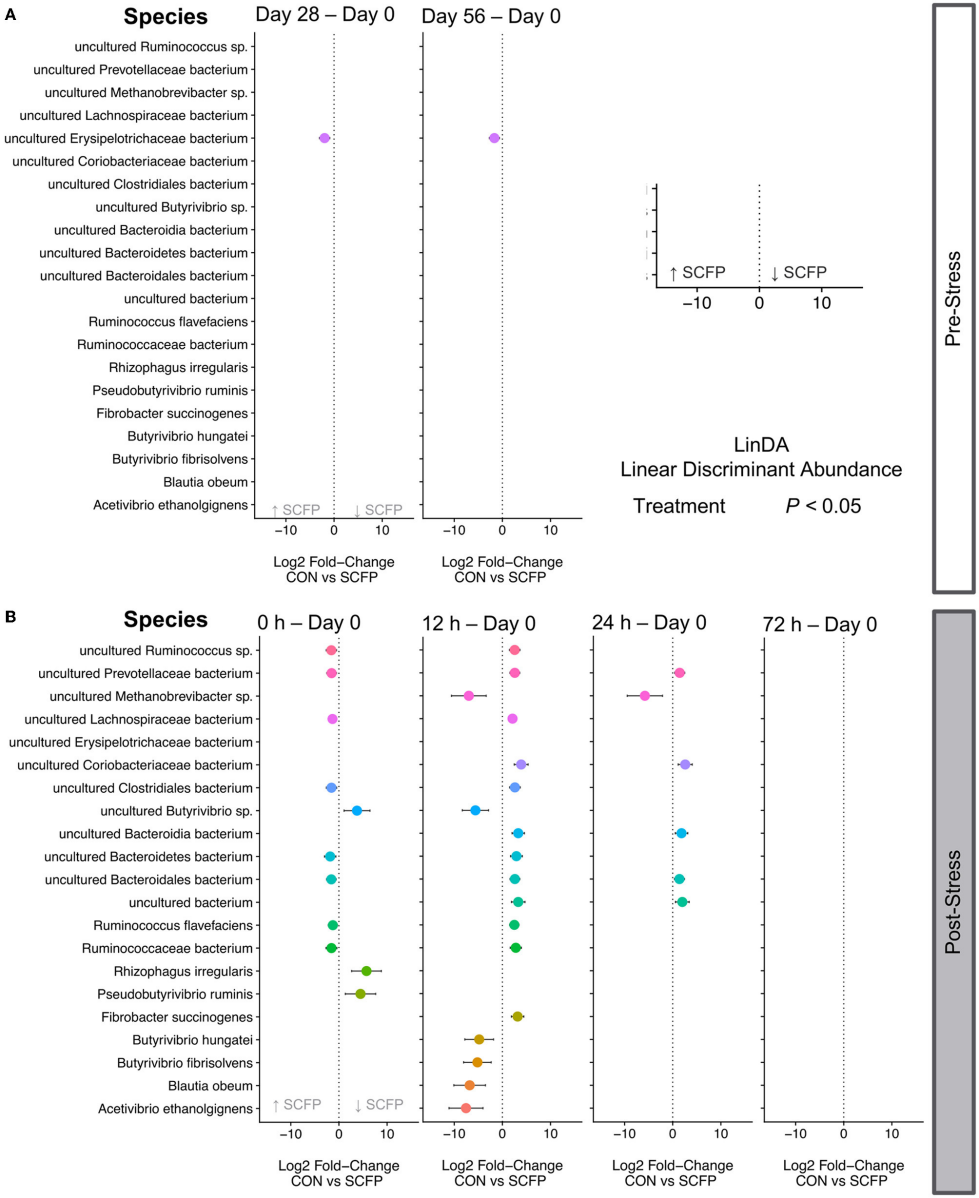
Differential abundances. The effect size (log2-fold change) is shown for each species, and only significantly different species are shown in each plot with their correspondent confidence interval. Statistical models included relative abundance as the dependent variable, horse as a random effect, treatment, timepoint, and their interactions as independent variables. Multiple hypothesis testing correction was performed with Benjamini Hochberg False Discovery Rate method. A negative fold change indicates an increase in relative abundance in SCFP compared to Control, and a positive fold indicates a decrease in relative abundance in SCFP compared to Control. **(A)** Differentially abundant species before stress. **(B)** Differentially abundant species after stress.

Treatment with SCFP significantly increased the abundances of *Erysipelotrichaceae* before stress challenge. In fact, this was the only significantly different taxa between Control and SCFP in the pre-challenge period (Figure 5A, panels 1 and 2), and SCFP treated animals had an overall positive log ratios throughout the entire study (Supplementary Figures).

Many more species were significantly differentially abundant after stress challenge particularly at times 0 and 12 h post-stress (Figure 5B). At time 0 h, eight species were significantly increased in the SCFP group compared to Control, and three species were significantly decreased (Figure 5B, panel 1). Statistically different species were observed between groups up to 24 h after stress (Figure 5B, panels 2 and 3), with no significantly different species observed at 72 h after stress (Figure 5B, panel 4).

### SCFP treated horses demonstrated more robust microbial functionality post-stress as compared to Control horses

**Figure 6:**
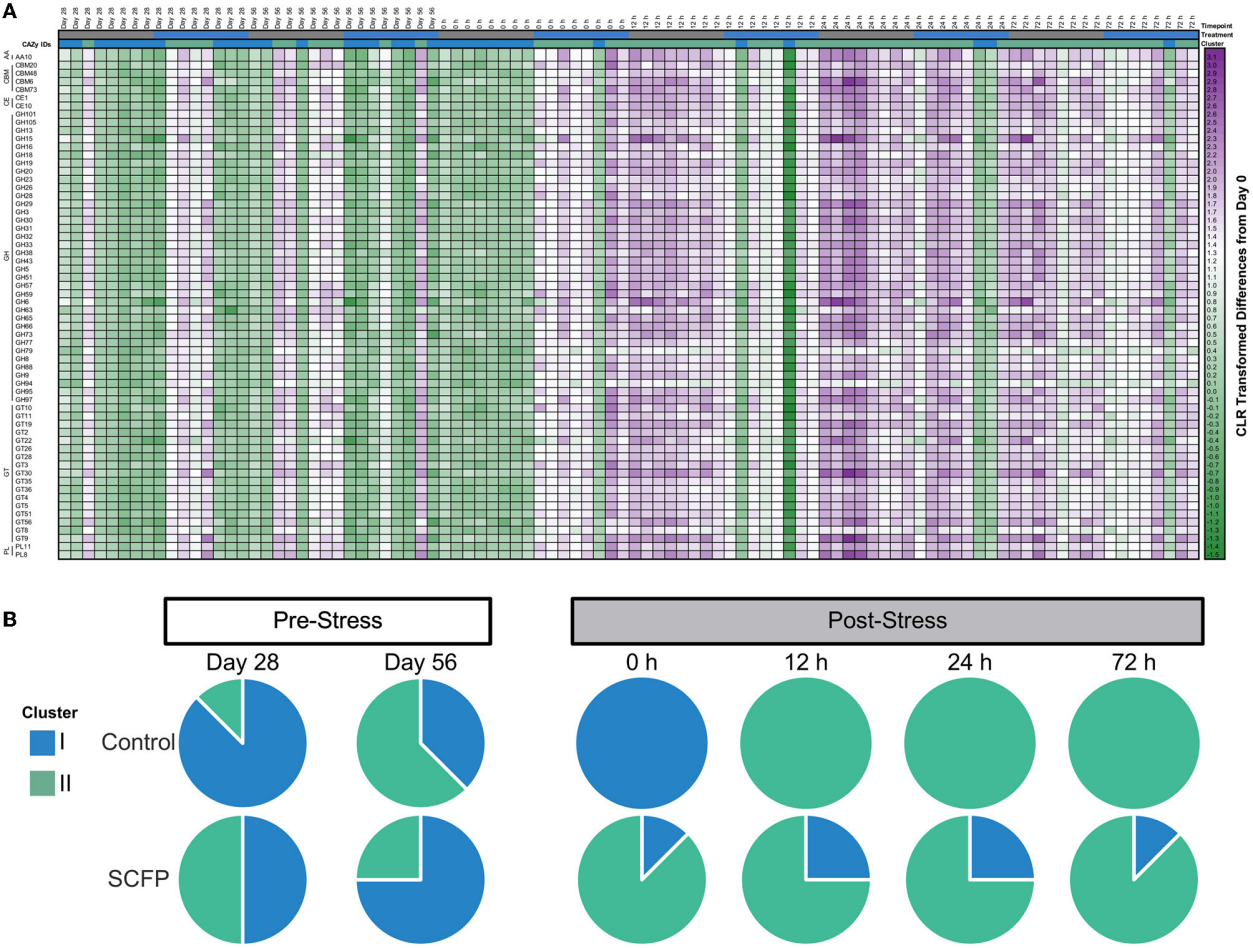
Functional potential is differentially impacted by treatment and stress in SCFP and Control horses. **(A)** Heatmap of centered log-ratio relative abundance of carbohydrate active enzymes (CAZy) families detected in fecal samples of horses. **(B)** Pie charts represent the presence of samples across different clusters for Control and SCFP groups across time. AA, Auxiliary Activity Family; CBM, Carbohydrate-Binding Module Family; CE, Carbohydrate Esterase Family; GH, Glycoside Hydrolase Family; GT, Glycosyl Transferase Family; PL, Polysaccharide Lyase Family.

Clustering analysis of the functional potential of the samples, measured by CAZy families, identified two major functional sample clusters (Figure 6A, clusters I and II). The CAZy families identified were Auxiliary Activity Family (AA), Carbohydrate-Binding Module Family (CBM), Carbohydrate Esterase Family (CE), Glycoside Hydrolase Family (GH), Glycosyl Transferase Family (GT), and Polysaccharide Lyase Family (PL). A more pronounced increase in CAZy families was observed after stress in Control horses compared to SCFP horses (Figure 6A). Similar to compositional clustering outcomes, Control and SCFP groups exhibited markedly different functional profiles following imposition of the stressor (Figure 6B), with Control horses demonstrating a switch to cluster I immediately after stress challenge, and again completely switching to cluster II from 12 to 72 h post-stress. Conversely SCFP treated horses displayed microbiome functional potential representatives of both clusters throughout the entire study period.

### Correlation networks reveal that post-stress microbial communities are more stable in SCFP treated horses

Overall, a smaller number of significant interactions were observed in the SCFP group compared to Control, particularly following stress (SCFP = 198 positive interactions and 30 negative interactions: Control = 304 positive interactions and 210 negative interactions; Supplementary Table 4). Treatment with SCFP resulted in a smaller number of significant species interactions overall (maximum of 310 interactions before challenge) while the Control group had a total of 520 interactions before challenge. Horses that received SCFP had fewer interactions in total compared to Control, both pre- and post-stress. While no difference was observed in the percentage of positive interactions before stress, SCFP treated horses had a substantially higher number of positive interactions after stress when compared to untreated Control horses (87 vs. 59%).

## Discussion

To evaluate the potential effect of supplementing horses under stressful conditions with a postbiotic, we sequenced the fecal metagenomes of 20 horses undergoing a previously established stress model that mimics prolonged transportation. The rationale that SCFP supplementation could lead to improved microbiome stability is based on recent reports of SCFP having a positive impact in other species undergoing stressful conditions (22, 24, 60–62). Here, we observed that untreated Control horses and treated (SCFP) horses presented very different microbiome trajectories upon stress, both in within- and between-sample diversity measurements. Moreover, a lower magnitude of changes was observed in the functional potential and microbial profile of SCFP horses vs. Control. Taken together, these observations led us to conclude that treatment with SCFP resulted in more robust and stable microbial profiles in horses after stress challenge.

Less variation in microbial and functional profiles were observed for SCFP compared to Control horses. This was noted in several of our analyses including Shannon diversity index, total number of microbial network interactions, percentage of positive network interactions, and microbial and functional clustering profiles, which was illustrated in heatmaps with Control horses having a higher degree of change than SCFP treated horses. These data led us to conclude that dietary SCFP supplementation results in a more stable and robust community that is less impacted by stress. Our findings are in agreement with Tun et al. (24) who observed that postbiotic treatment tends to stabilize the microbiota of cows in a subclinical acidosis challenge. In that study authors concluded that SCFP supplementation attenuated the impacts of subacute ruminal acidosis on the composition and functionality of the rumen microbiome. Taken together, our results and those from others leads us to hypothesize that SCFP treatment results in a microbial community that is more robust (defined as resistance against change) in responding to stress. This hypothesis is corroborated by observations in our correlation network analyses, where the number of connections generally decreased with SCFP, but the percentage of positive interactions increased, indicating that a leaner (defined as a community with fewer connections among members but with a larger number of positive connections) and more connected microbial community in SCFP horses.

Our results are also in agreement with other studies that demonstrated that postbiotic supplementation is associated with improved microbiome balance which has been translated into host health in many species (23, 60, 62, 63). In horses, postbiotic supplementation resulted in increased relative abundances of fibrolytic bacteria (64) and attenuated exercise-induced stress markers (28). Additionally, Lucassen et al. showed that postbiotic-treated horses have more efficient response to vaccination (23). In contrast, a recent study by the same group evaluating SCFP supplementation did not identify significant alterations in the fecal microbiota of thoroughbred racehorses (27). Possible explanations for this disparity are the use of a lower resolution technique (16S rRNA gene sequencing) and the smaller dataset (11 horses) in that study when compared to our study, which used shotgun metagenomics to analyze the fecal microbiome of 20 horses. Additionally, those authors reported a high degree of horse-dependent effects of treatment, which can be attributed to high horse-to-horse variability.

The potential effects of stress and SCFP treatment on CAZy families was evaluated due to the importance and dependence of the horse on the degradation of structural carbohydrates of forages by gut microbiome for health and wellbeing. We observed that SCFP stabilized the composition and functionality of the hindgut microbial community. This was observed particularly immediately after stress relief (0 h) where lower CAZy abundance was observed in Control horses (light green in most cases) while abundances remained relatively unchanged or increased in SCFP (with the exception of one horse). At 12 h post-stress, Control horses displayed a dramatic switch in functional profile, with most showing increased relative abundances as illustrated in the heat maps in Figure 6. The small sample size of this study precludes us from making further statements regarding the functional potential, but what is evident from this study is that larger swings in relative abundances of CAZy families were associated with Control horses when compared to treated horses throughout the entire post-stress period. Additionally, a strong horse-to-horse effect was observed, indicating that treatment effect is highly dependent on the animal. These findings are similar to those of Lucassen et al. (27) who observed a high degree of horse-to-horse variability in their study of the equine microbiome of horses fed a postbiotic.

The bacteria identified in our study are in agreement with previous reports of healthy equine gut microbiomes, with a composition that is mainly dominated by fibrolytic bacteria (8, 9, 65). It is important to highlight that we chose a very strict threshold for taxa selection for statistical comparisons between treatment groups. Specifically, to be included in the statistical analysis, a microbial species had to be present in at least 75% of all samples. This was a deliberate choice to decrease the chances for spurious findings due to multiple hypothesis testing.

Here, we identified that SCFP treatment significantly impacted the relative abundance of *Erysipelotrichaceae* before stress, with a small, but significant increase in SCFP treated horses compared to Control horses. Biddle and colleagues also observed significant temporal changes in *Erysipelotrichaceae* in obese horses (66), and this family had previously been identified as part of the core microbial community of horse feces (67). However, little is known about the role of this species in the horse gut and diverging evidence has been presented about the role of *Erysipelotrichaceae* in other organisms, with varying levels of *Erysipelotrichaceae* reported in murine and human studies of disease (12, 68).

Immediately after stress 11 bacteria were identified to be significantly different albeit with very small effect sizes. From those, eight were increased in the SCFP group and three were increased in the Control group, with relatively higher effect sizes when compared to species increased in SCFP horses. Microorganisms significantly increased in Control horses included one uncultured *Butyrivibrio* species, *Pseudobutirivibrio ruminis*, and *Ryzophagus irregularis*. We identified *Ryzophagus irregularis*, an arbuscular mycorrhizal fungus that is common in plants, and which (69) has not been previously reported in the horse gastrointestinal tract. Given the presence of this organism in many plant species, and the plant-based diet of horses, this finding is not completely surprising.

A larger number of significantly different species were observed at 12 h post stress. Out of 18 significantly different species, six had relatively high effect sizes and were increased in SCFP horses. These included three *Butyrivibrio* species in addition to *Blautia, Acetivibrio*, and *Methanobrevibacter*, which were found to have significantly higher relative abundances in SCFP treated horses 12 h after stress. *Butyrivibrio* are very versatile bacteria and encode a variety of enzymes to hydrolyze complex carbohydrates (70, 71). They have been reported to carry many genes encoding glycoside hydrolases (GH) that are involved in carbohydrate fermentation and butyrate production. Likewise, *Blautia* and *Acetivibrio* are also fiber fermenters (8). In agreement with increases with *Butyrivibrio* species, in our functional annotation analyses, 36 out of 62 enzymes found to be significantly different in the present study encode for glycoside hydrolases. Lastly, *Methanobrevibacter* was also identified to be increased in the SCFP group at 12 and 24 h post stress. The presence of methanogenic archaea in the horse gut has been previously reported, and the diversity of methane producers in the horse gut is believed to be high (8, 72).

Despite the wealth of data collected as part of this project we acknowledge that a sample size of 20 horses is relatively small. It was further substantiated that horse intrinsic factors impacted the response to stress or treatment, as it could be observed by single animals behaving differently than the remainder of the group at a given timepoint. In fact, horse-to-horse variability has been well-documented in immune parameters in horses subjected to this model of stress (33). Raidal et al. observed varying degrees of change in white cell count, neutrophil count, and total bacterial numbers in six horses subjected to prolonged head elevation (33). This added variability might have confounded our analyses and precluded us from identifying strong signals. Nevertheless, animals are different and thus further research should account for animal-to-animal changes and perhaps quantify the effect in terms of microbiome changes within an animal. Even with the relatively high heterogeneity of this dataset we were able to identify a clear overall signal that treatment with SCFP tends to promote microbiome robustness and stability after stress, as we observed in measures of alpha and beta diversity, as well as bacterial and functional profiles, and bacteria interaction dynamics.

This study adds to the body of knowledge regarding the beneficial impacts of postbiotic administration to horses undergoing stressful situations. While the specific mechanisms by which this robustness and stability are imparted in an equine's gut microbiome by postbiotic administration are not fully elucidated, studies in other species suggested that potential underlying mechanisms by which postbiotic supplementation led to improved health include effects in immunomodulatory pathways (73) and improved microbiome composition and functionality (24). From an immune perspective, animals receiving SCFP seem to be primed to respond with elevated (magnitude of response) and accelerated (speed of response) cytokine production when a threat is detected (61, 62). Additionally, at the site of challenge, increased phagocytic activity and killing ability of white blood cells and reduced activation of inflammatory system leads to a reduction in localized inflammation, and potentially immunopathology in SCFP supplemented animals (62, 74). From the microbiome perspective, ruminant studies have shown that SCFP supplementation boosts the abundances of influential members of the microbiome which promote richness and diversity, and hence, functionality of the microbiome resulting in increased VFA production and improved energetic efficiency of rumen fermentation (24, 75). Therefore, it can be speculated that the dual action of SCFP postbiotic *via* immunomodulatory pathways and optimized microbiome functionality increases robustness of animals against a wide range of infectious and metabolic stressors.

Our results indicate that prophylactic supplementation with a yeast-derived postbiotic might be a beneficial strategy for horses prior to exposure to stress. This exploratory study is limited in the ability to draw mechanistic conclusions on the effects of SCFP in horses subjected to a stress model. We observed a lower degree of change both in microbial diversity and functional profile of horses fed SCFP when compared to Control. Mechanistically, having a more robust and stable microbiome plausibly results in less opportunity for pathogen colonization and better health maintenance. Postbiotics have been demonstrated to have positive impacts in several species, and further research into the mechanisms by which these beneficial effects occur is warranted.

## Data availability statement

The datasets presented in this study can be found in online repositories. The names of the repository/repositories and accession number(s) can be found at: https://www.ncbi.nlm.nih.gov/, bioproject PRJNA788958.

## Ethics statement

The animal study was reviewed and approved by Institutional Animal Care and Use Committee at the University of Florida in Gainesville, FL (#201810324).

## Author contributions

LW designed the animal experiment. EK and BK designed the microbiome study. MT performed the animal experiment and collected samples. MS performed sequencing and bioinformatics. AC performed statistical analyses. EG, EK, and SN interpreted the data. EG and EK prepared the first draft. All authors read and approved the final manuscript.
