# Supplementary material for: Saccharomyces cerevisiae fermentation product improves robustness of equine gut microbiome upon stress
Source: Front Vet Sci. 2023 Feb 24;10:1134092. doi: 10.3389/fvets.2023.1134092 (PMC9998945; doi:10.3389/fvets.2023.1134092)
Supplement: Supplementary file 1 [file Data_Sheet_1.zip › Supplementary Figures.PDF]

Supplementary Figures

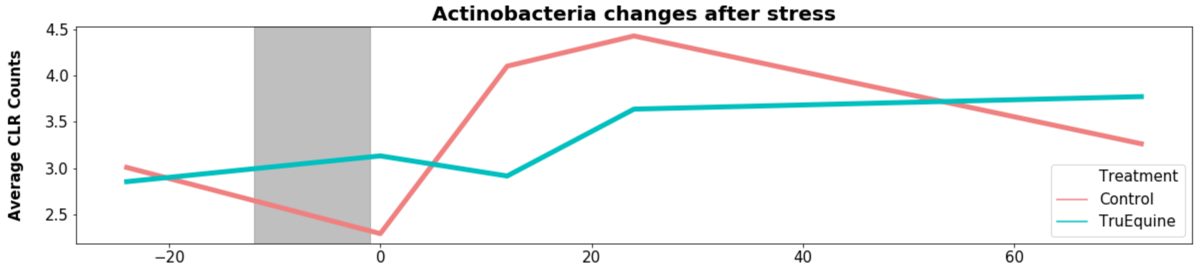
Actinobacteria changes after stress
Average CLR Counts
4.5
4.0
3.5
3.0
2.5
−20
0
20
40
60
Treatment
Control
TruEquine

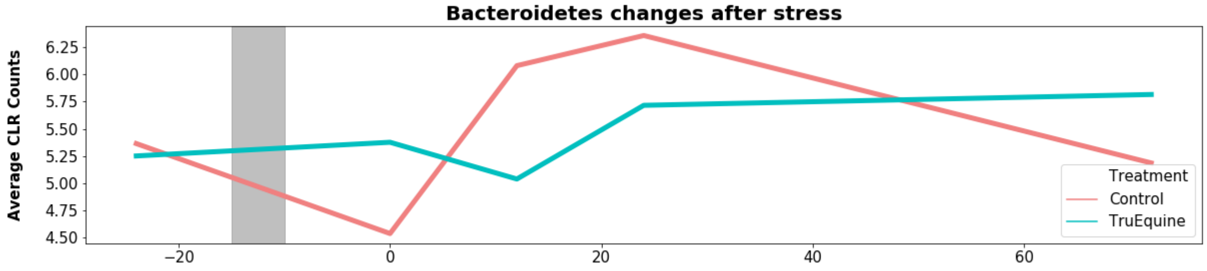
Bacteroidetes changes after stress
Average CLR Counts
6.25
6.00
5.75
5.50
5.25
5.00
4.75
4.50
−20
0
20
40
60
Treatment
Control
TruEquine

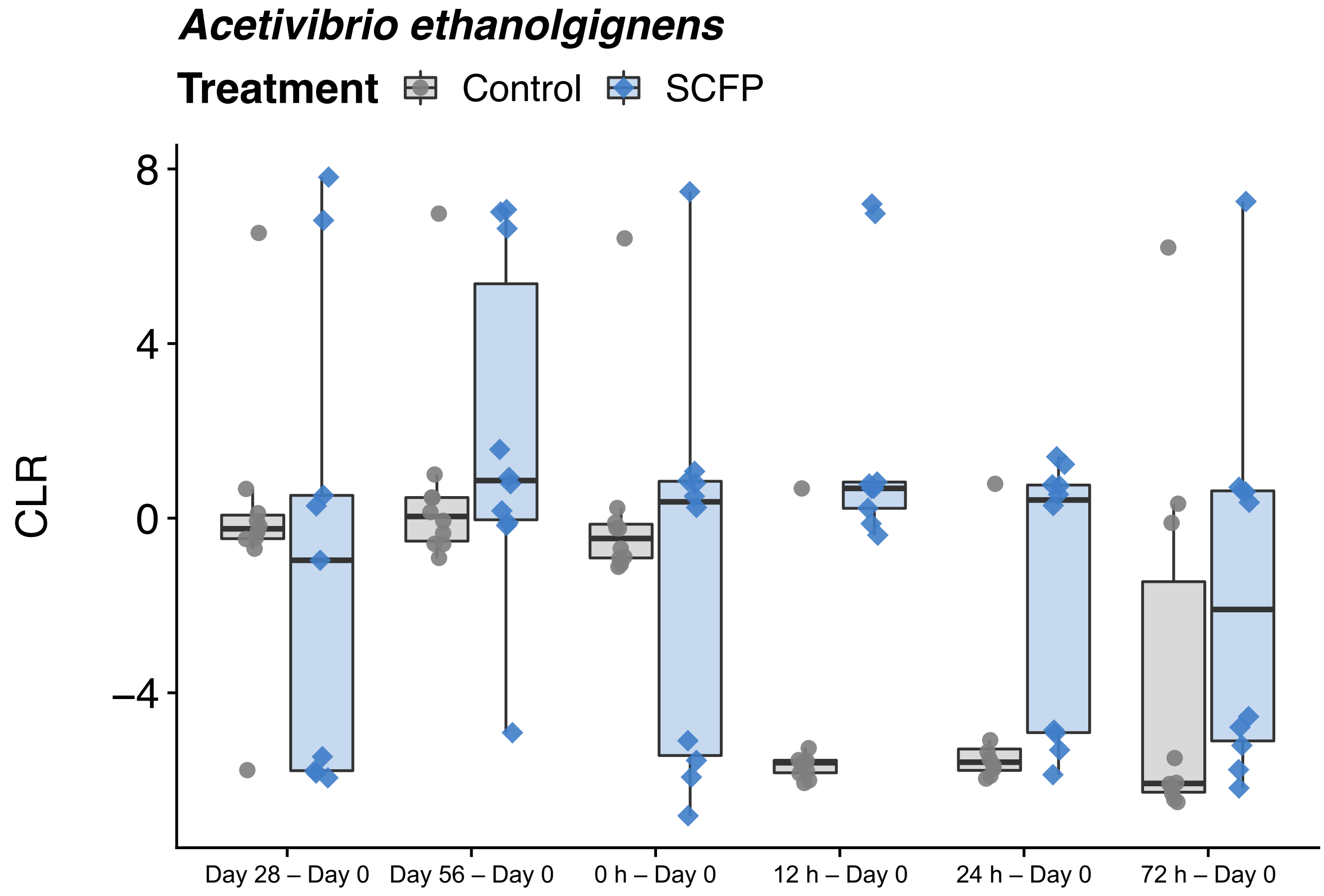
Acetivibrio ethanolgignens
Treatment
Control
SCFP
8
4
0
−4
CLR
Day 28 – Day 0
Day 56 – Day 0
0 h – Day 0
12 h – Day 0
24 h – Day 0
72 h – Day 0

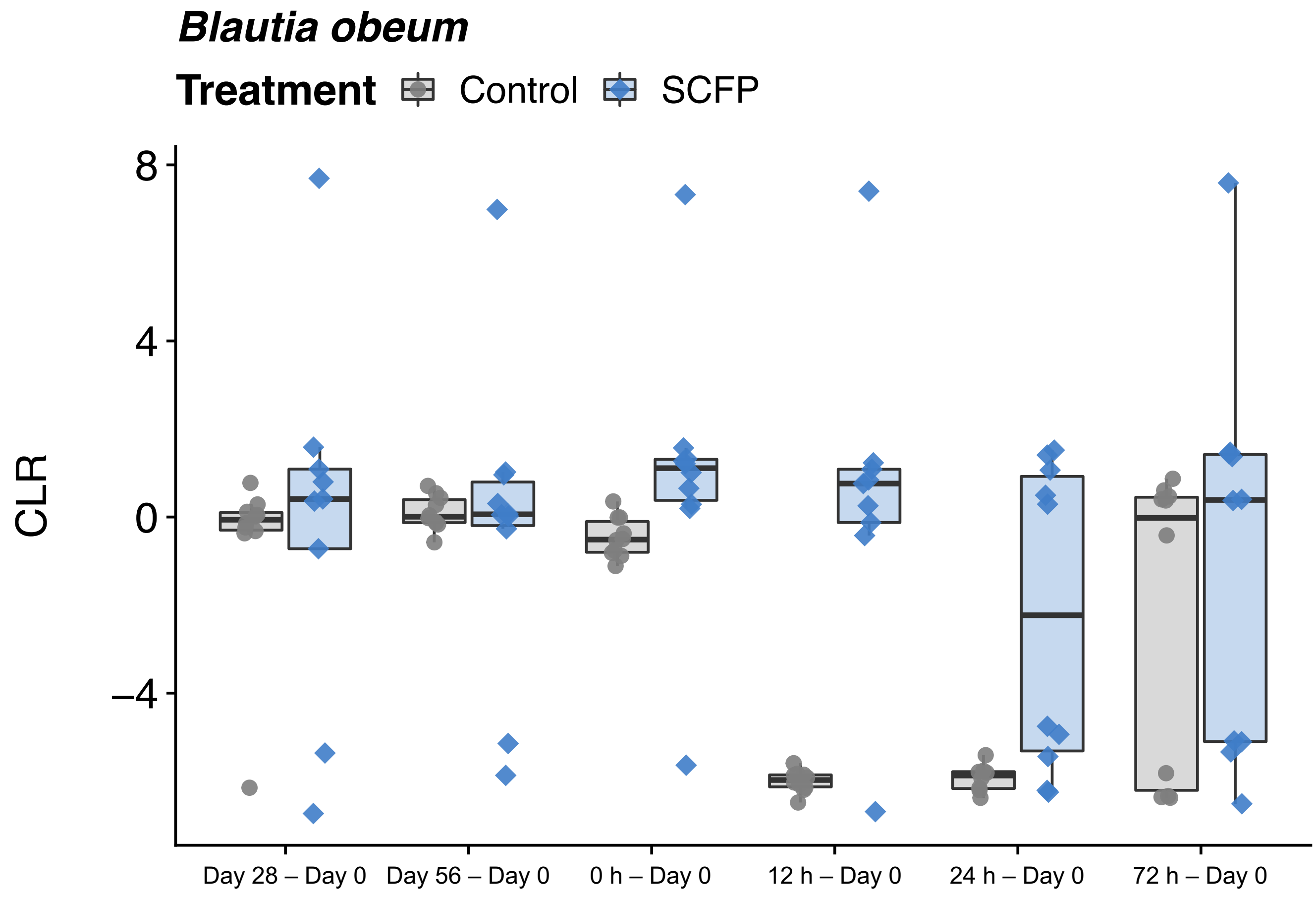
Blautia obeum
Treatment
Control
SCFP
8
4
0
−4
CLR
Day 28 – Day 0
Day 56 – Day 0
0 h – Day 0
12 h – Day 0
24 h – Day 0
72 h – Day 0

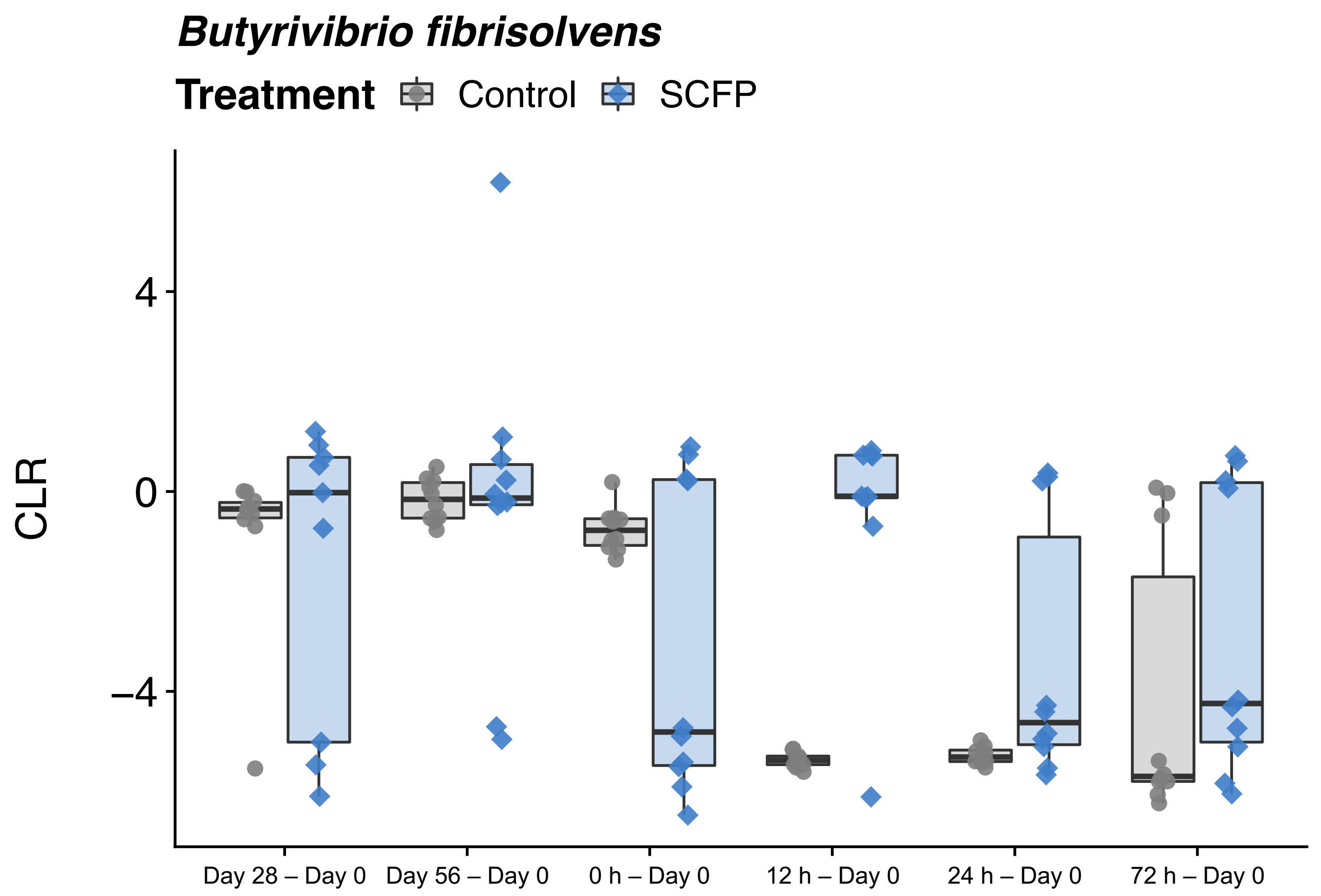

Butyrivibrio fibrisolvens
Treatment
Control
SCFP
CLR
4
0
−4
Day 28 – Day 0
Day 56 – Day 0
0 h – Day 0
12 h – Day 0
24 h – Day 0
72 h – Day 0

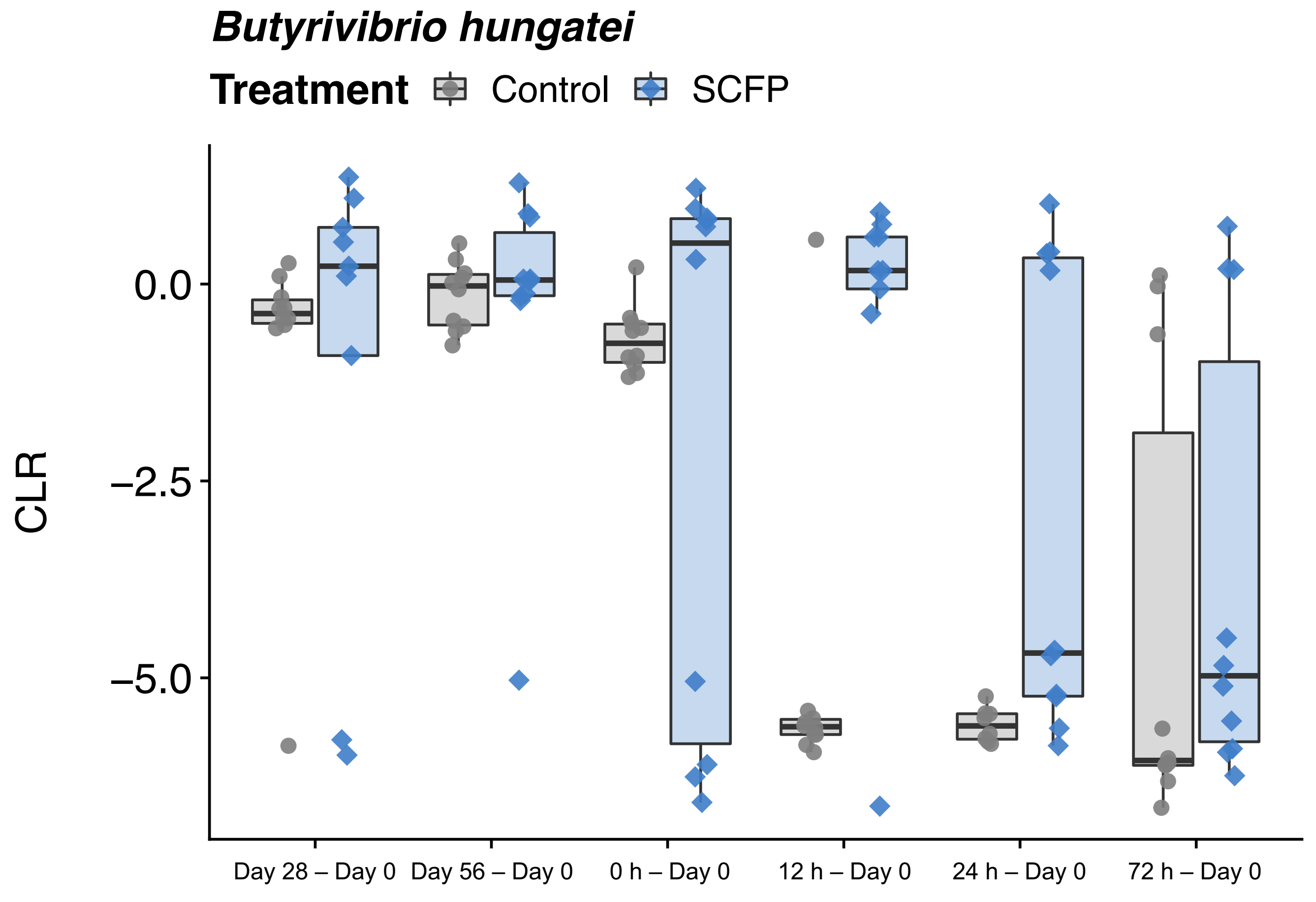

Butyrivibrio hungatei
Treatment
Control
SCFP
CLR
0.0
−2.5
−5.0
Day 28 – Day 0
Day 56 – Day 0
0 h – Day 0
12 h – Day 0
24 h – Day 0
72 h – Day 0

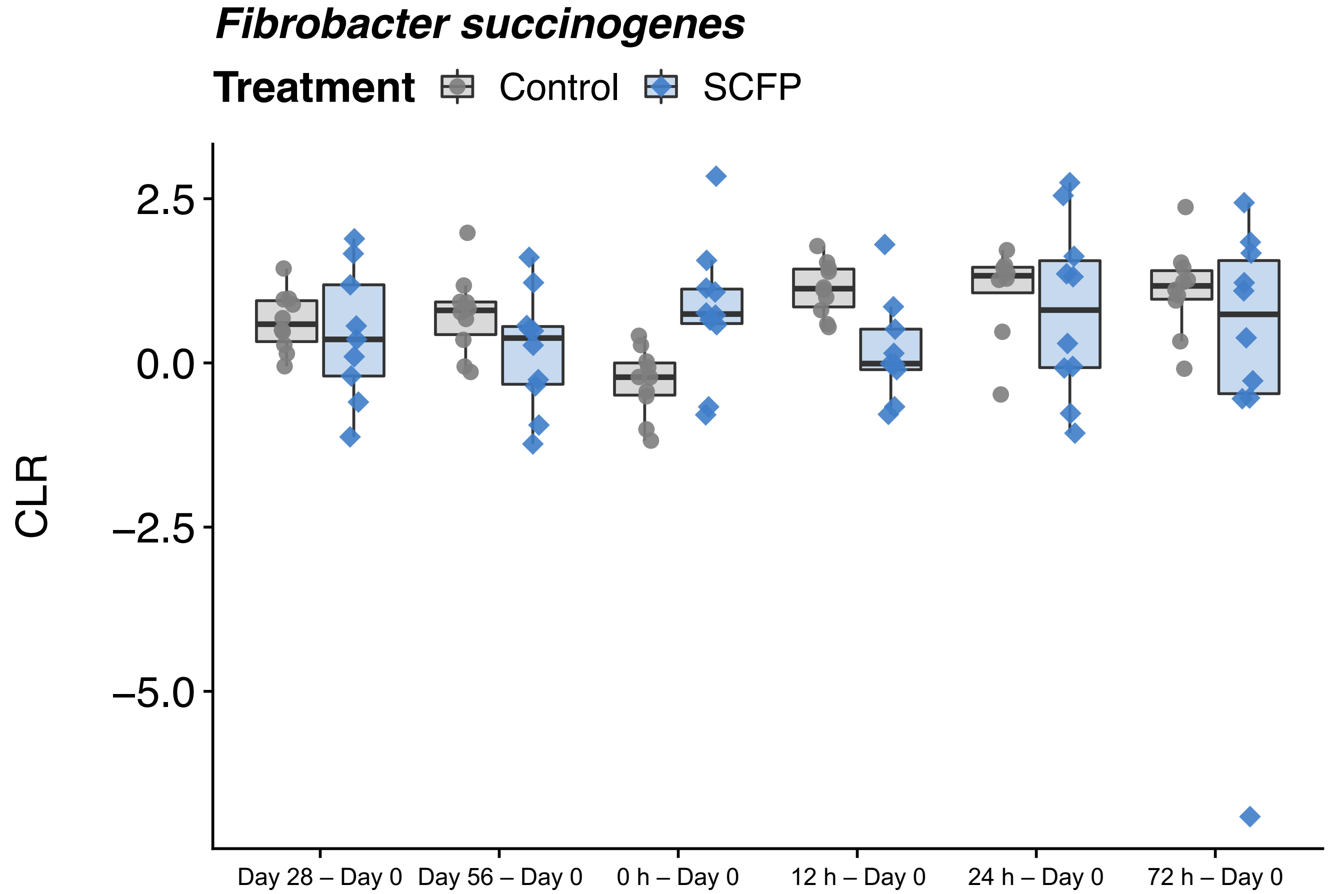

Fibrobacter succinogenes
Treatment
Control
SCFP
CLR
2.5
0.0
−2.5
−5.0
Day 28 – Day 0
Day 56 – Day 0
0 h – Day 0
12 h – Day 0
24 h – Day 0
72 h – Day 0

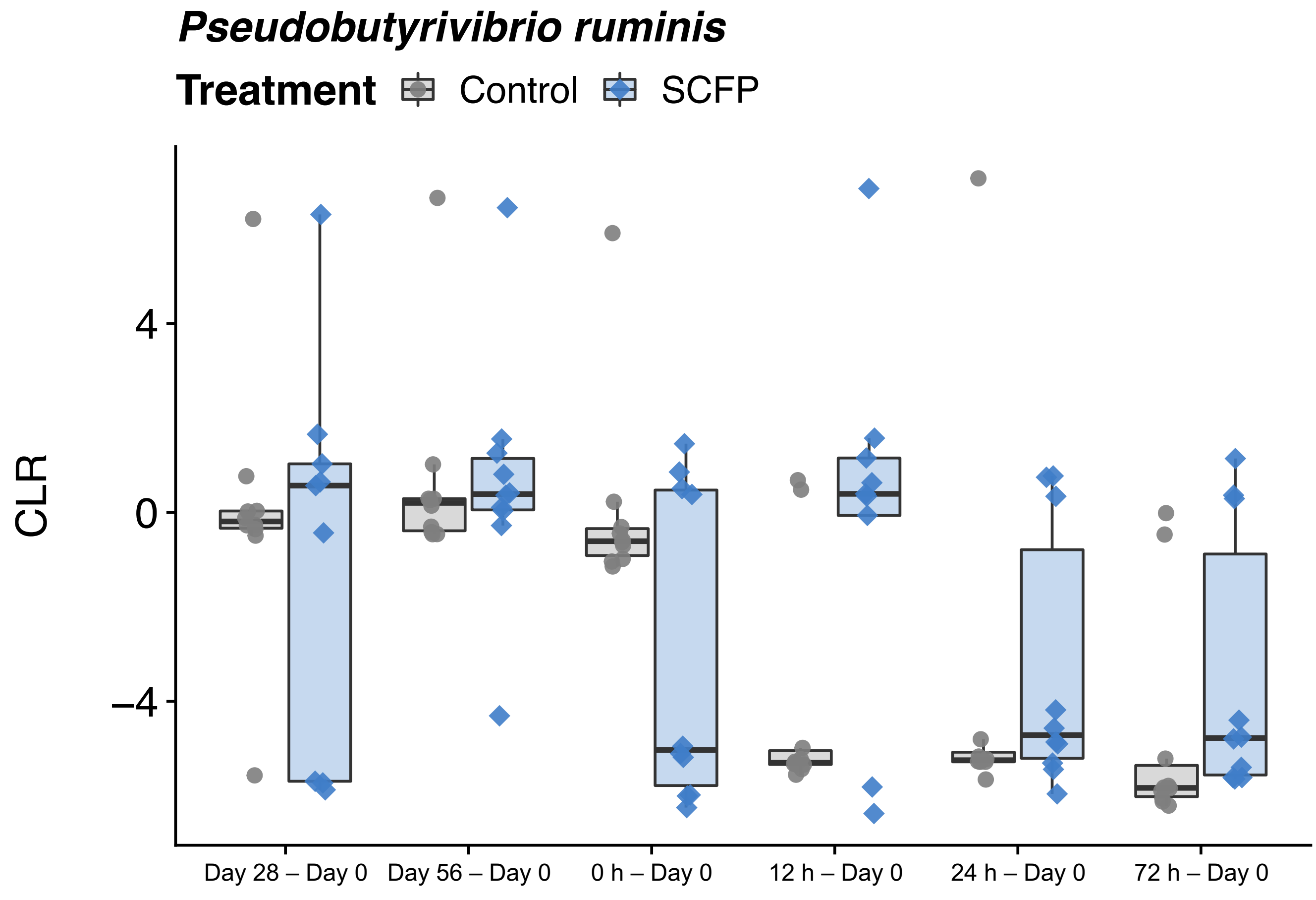

Pseudobutyrivibrio ruminis
Treatment
Control
SCFP
CLR
4
0
−4
Day 28 – Day 0
Day 56 – Day 0
0 h – Day 0
12 h – Day 0
24 h – Day 0
72 h – Day 0

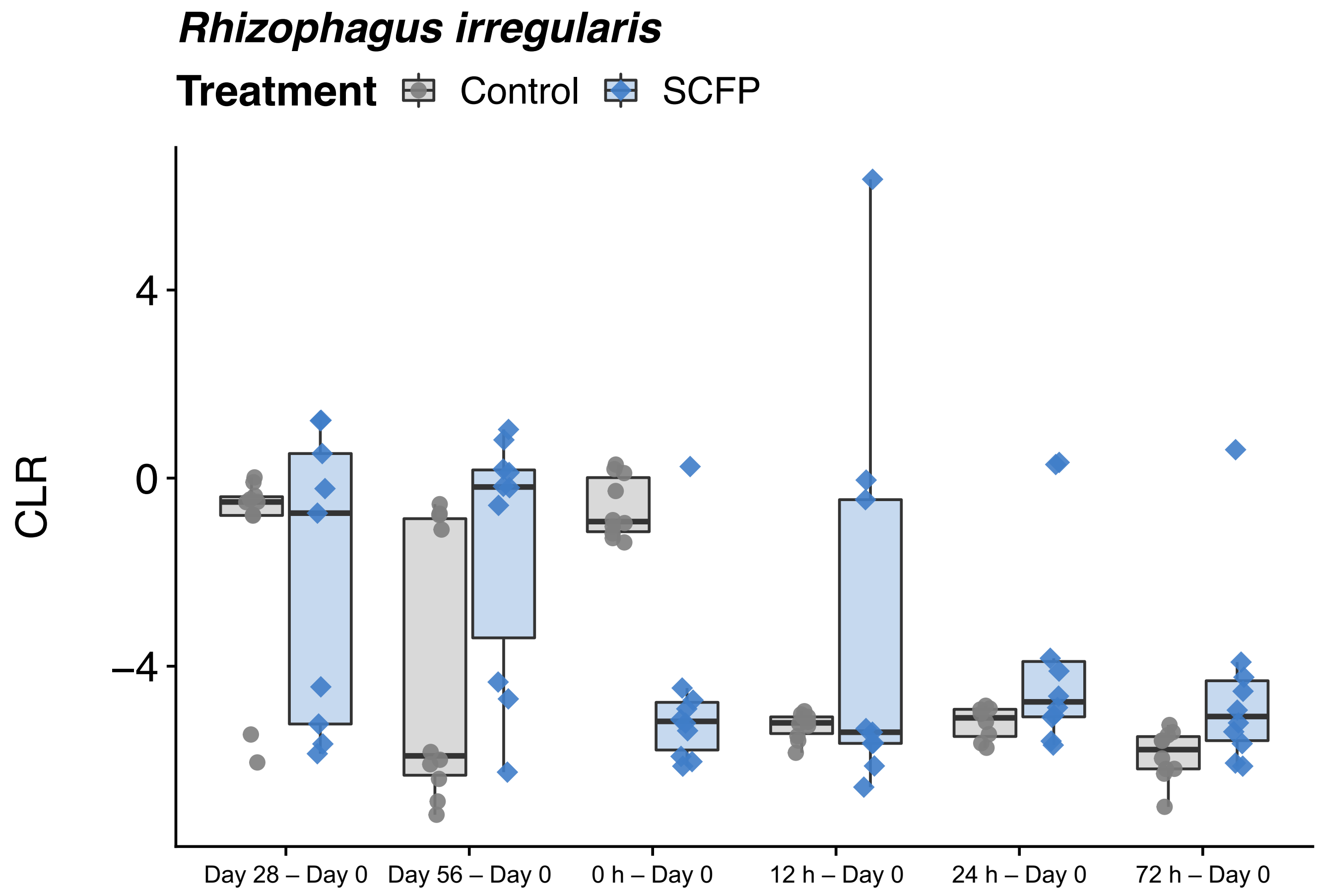

Rhizophagus irregularis
Treatment
Control
SCFP
CLR
4
0
−4
Day 28 – Day 0
Day 56 – Day 0
0 h – Day 0
12 h – Day 0
24 h – Day 0
72 h – Day 0

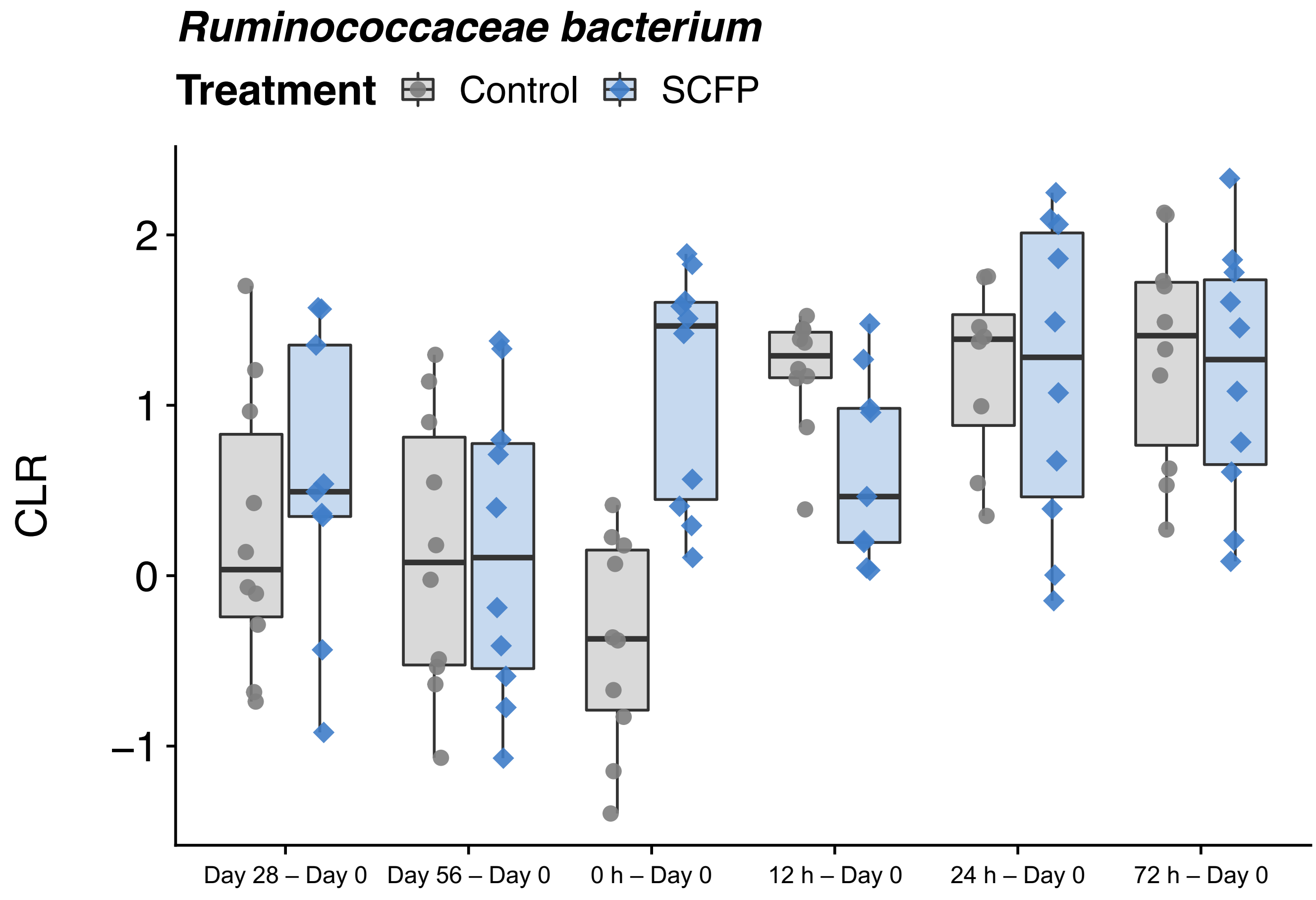

Ruminococcaceae bacterium
Treatment
Control
SCFP
CLR
2
1
0
−1
Day 28 – Day 0
Day 56 – Day 0
0 h – Day 0
12 h – Day 0
24 h – Day 0
72 h – Day 0

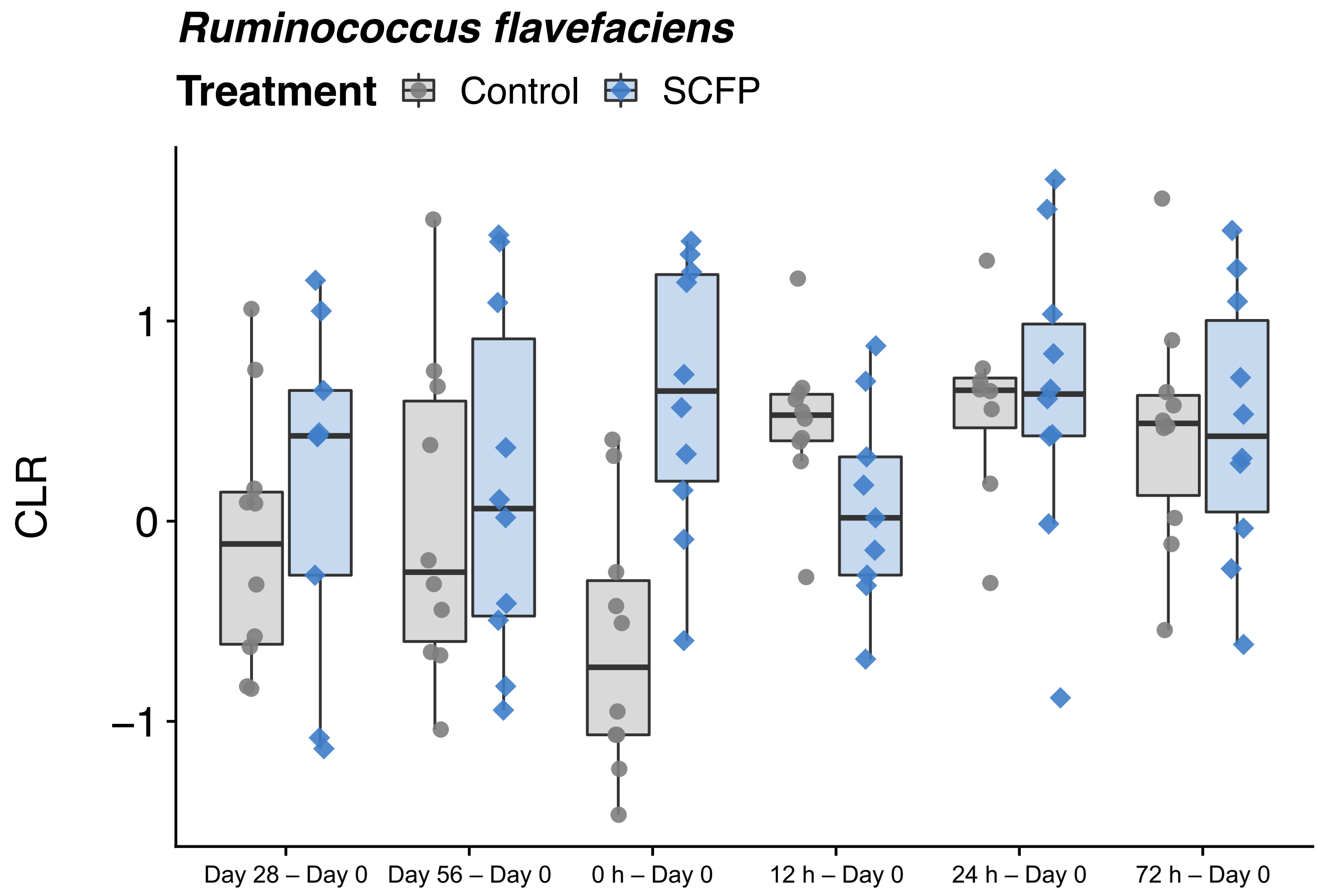
Ruminococcus flavefaciens
Treatment
Control
SCFP
CLR
1
0
−1
Day 28 – Day 0
Day 56 – Day 0
0 h – Day 0
12 h – Day 0
24 h – Day 0
72 h – Day 0

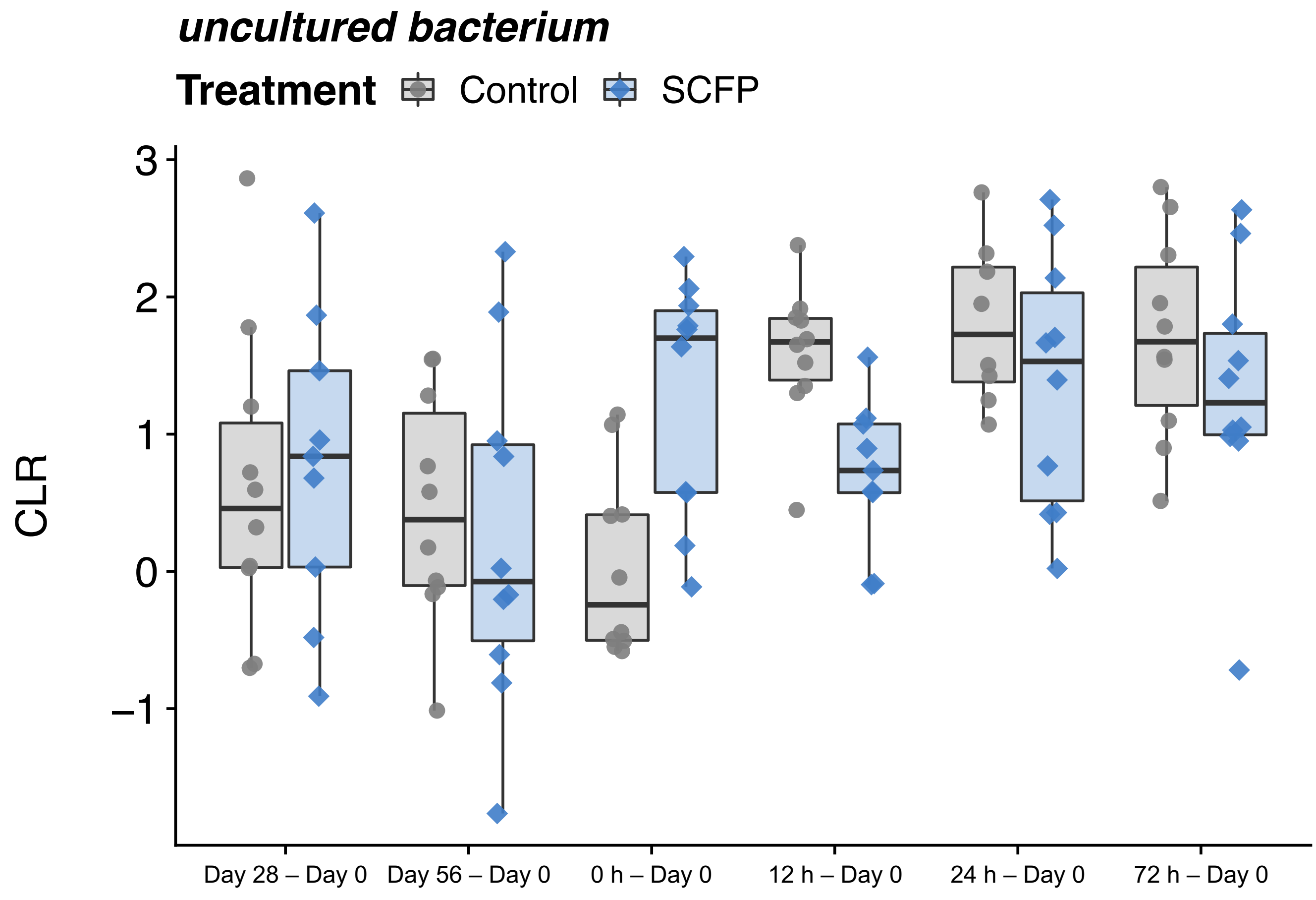
uncultured bacterium
Treatment
Control
SCFP
CLR
3
2
1
0
−1
Day 28 – Day 0
Day 56 – Day 0
0 h – Day 0
12 h – Day 0
24 h – Day 0
72 h – Day 0

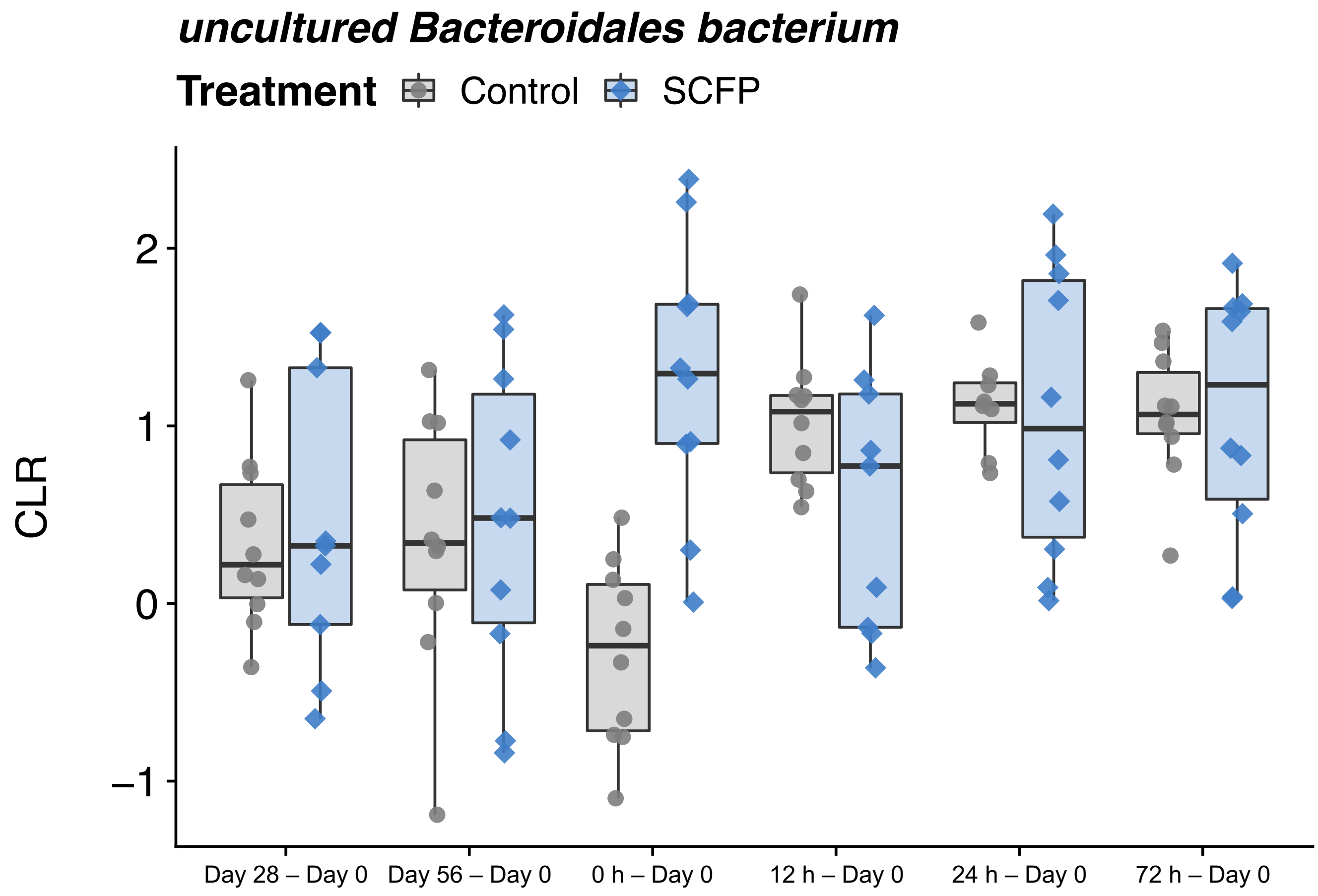
uncultured Bacteroidales bacterium
Treatment
Control
SCFP
CLR
2
1
0
−1
Day 28 – Day 0
Day 56 – Day 0
0 h – Day 0
12 h – Day 0
24 h – Day 0
72 h – Day 0

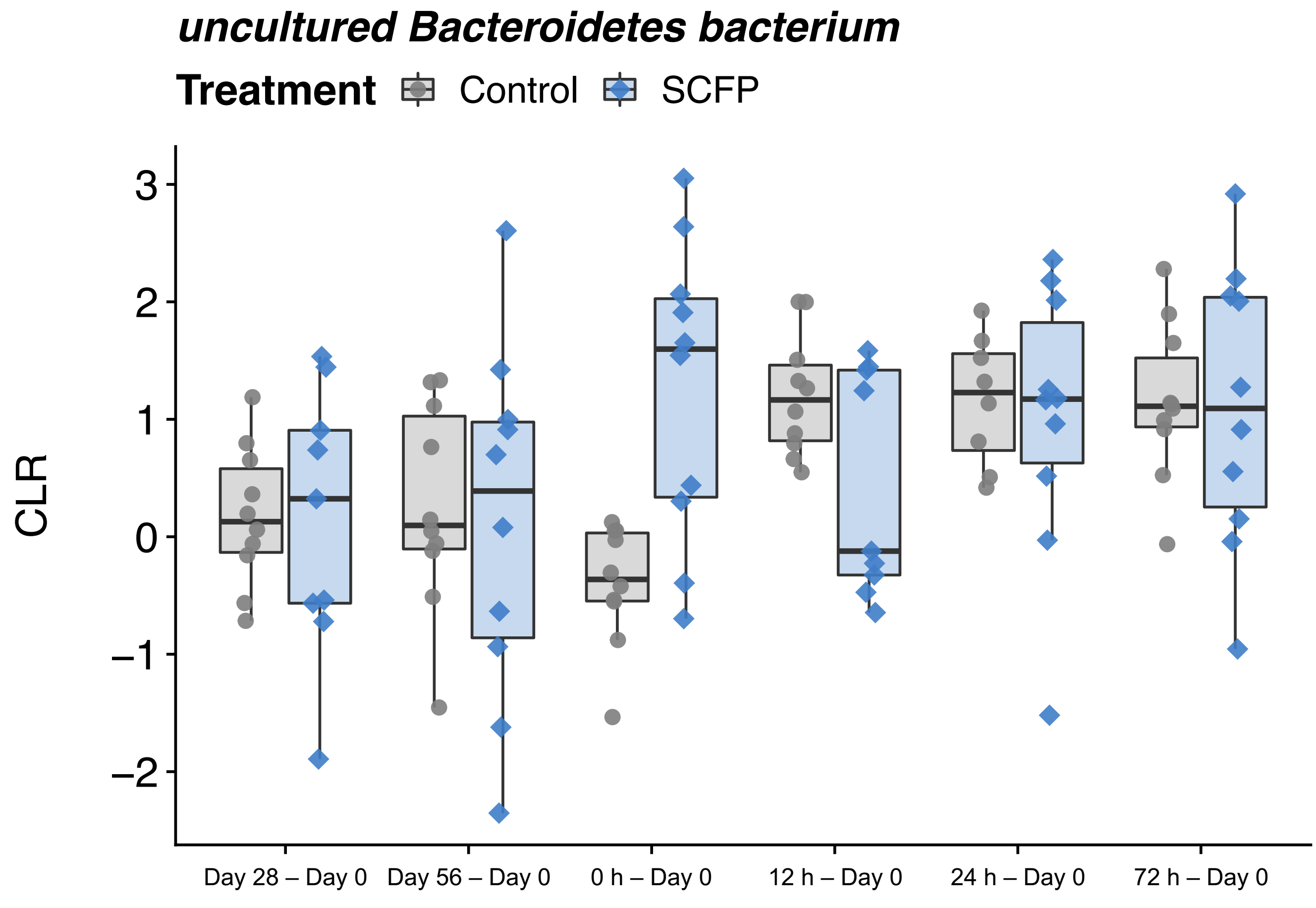
uncultured Bacteroidetes bacterium
Treatment
Control
SCFP
CLR
3
2
1
0
−1
−2
Day 28 – Day 0
Day 56 – Day 0
0 h – Day 0
12 h – Day 0
24 h – Day 0
72 h – Day 0

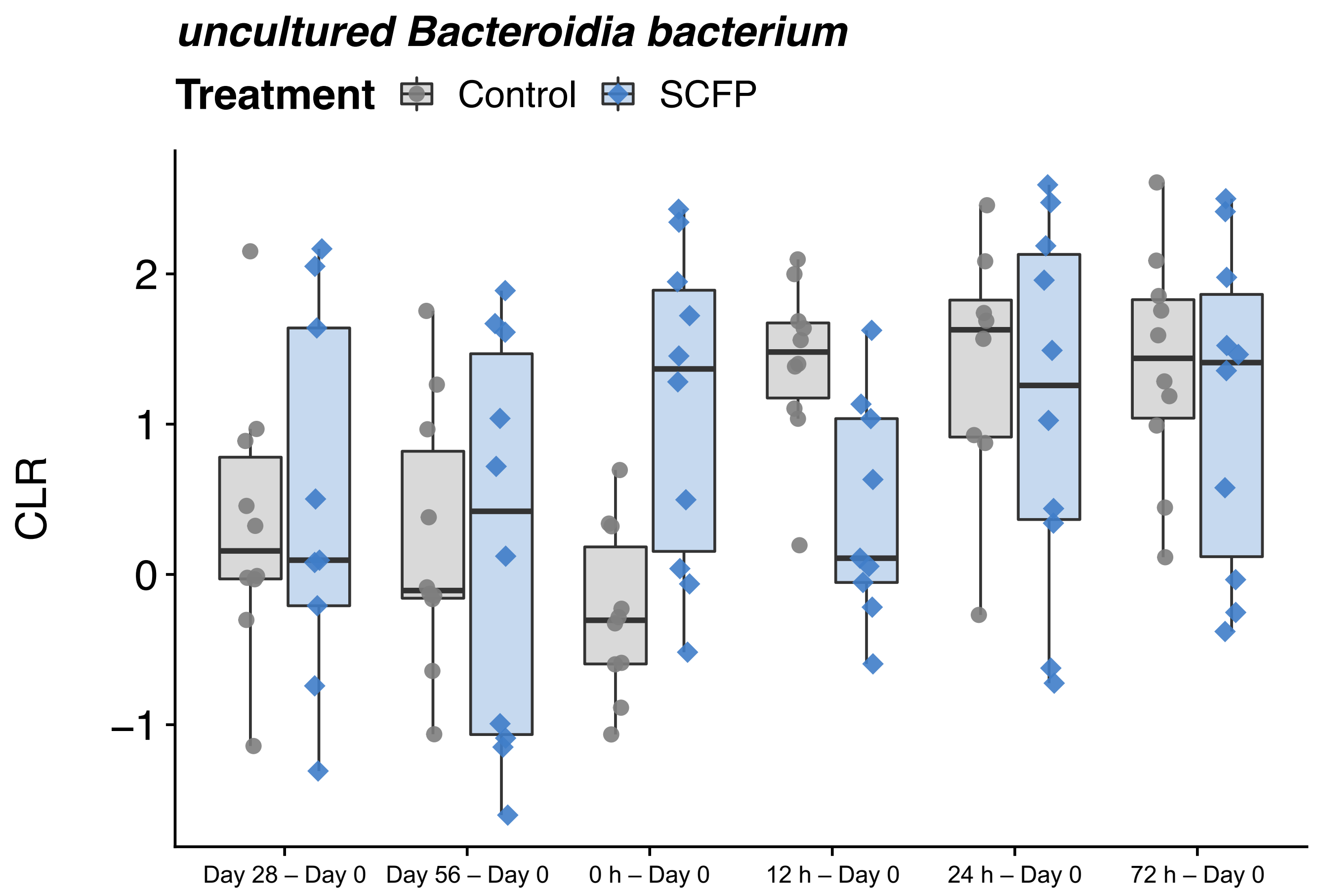
uncultured Bacteroidia bacterium
Treatment
Control
SCFP
CLR
2
1
0
−1
Day 28 – Day 0
Day 56 – Day 0
0 h – Day 0
12 h – Day 0
24 h – Day 0
72 h – Day 0

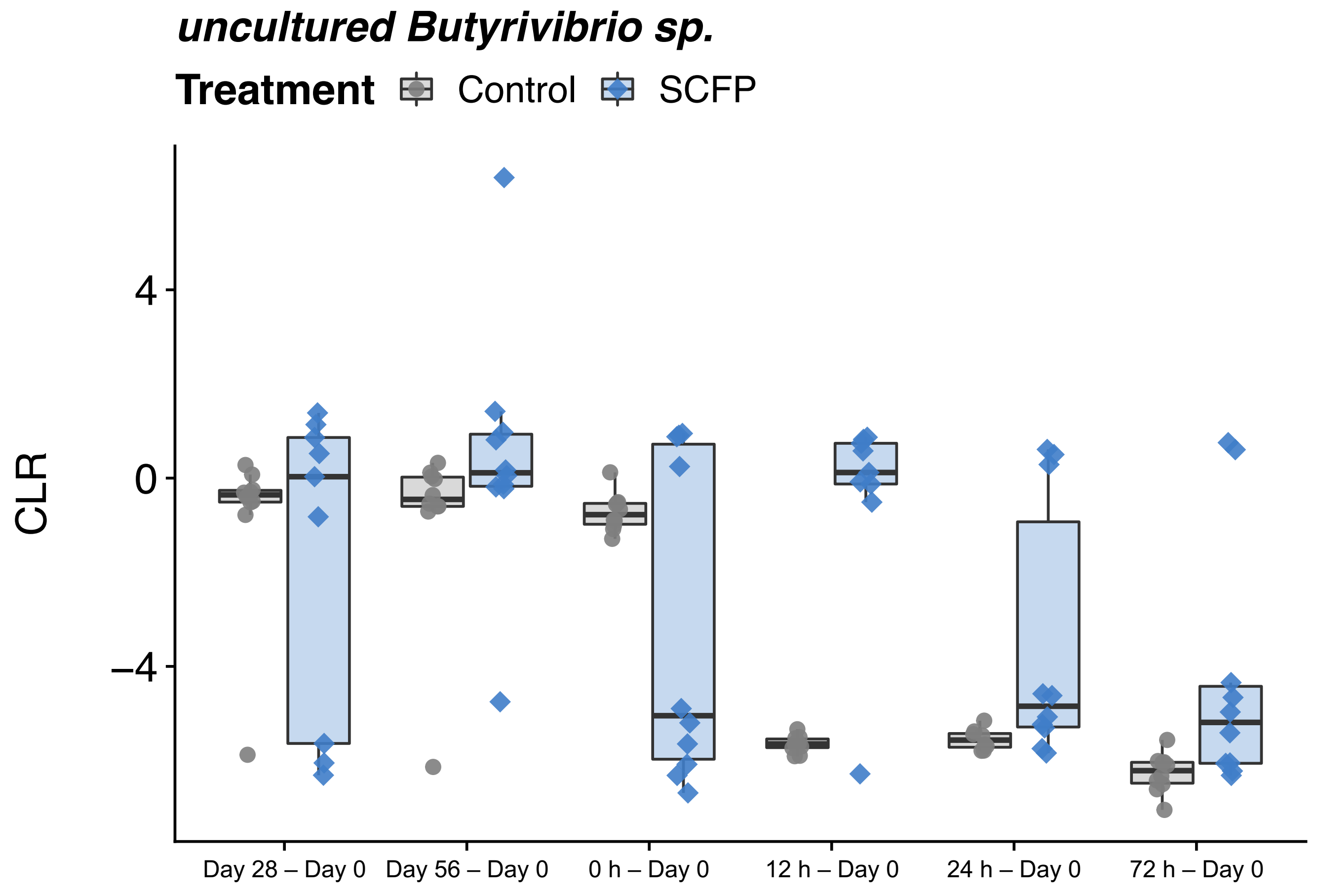
uncultured Butyrivibrio sp.
Treatment
Control
SCFP
CLR
4
0
−4
Day 28 – Day 0
Day 56 – Day 0
0 h – Day 0
12 h – Day 0
24 h – Day 0
72 h – Day 0

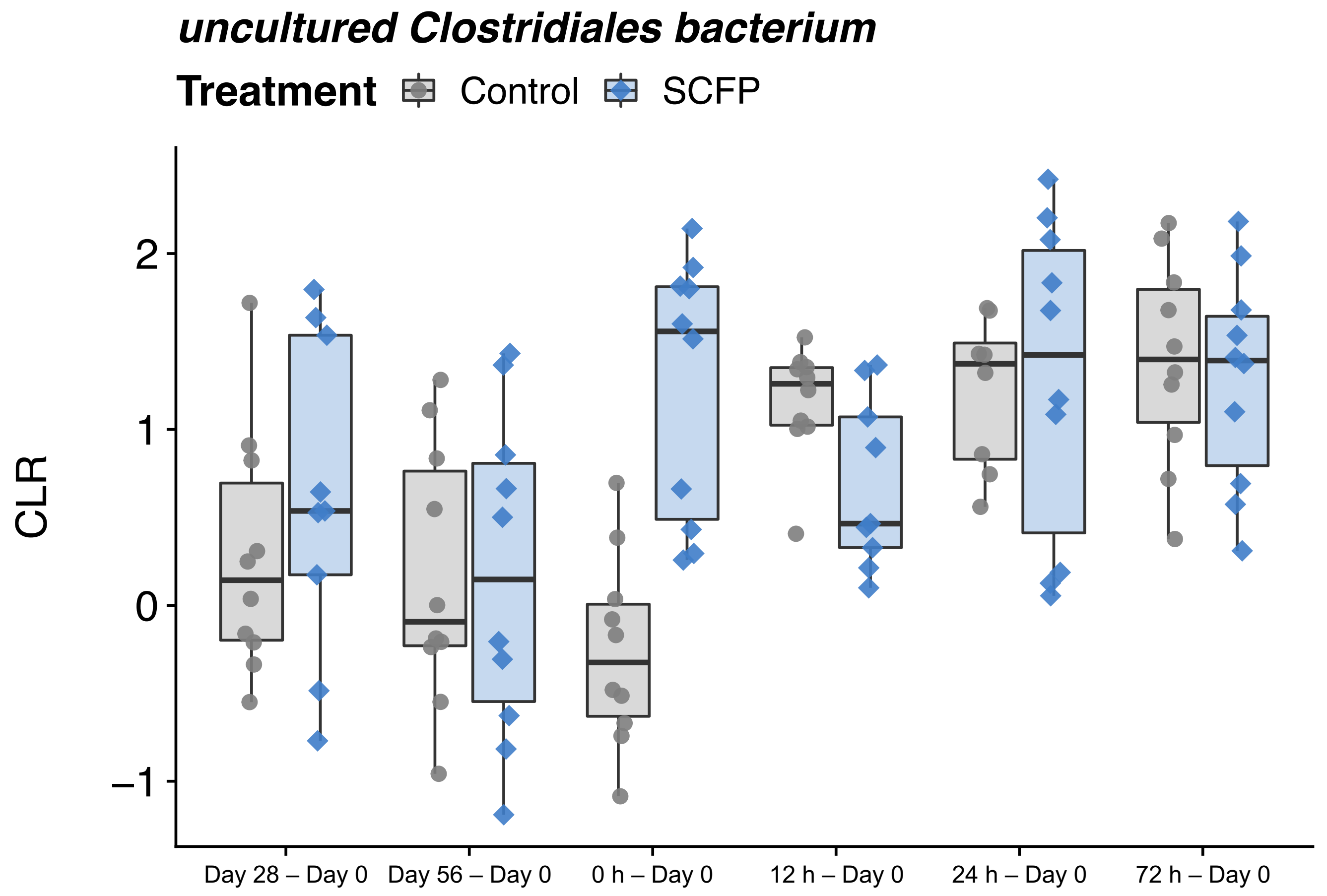
uncultured Clostridiales bacterium
Treatment
Control
SCFP
CLR
2
1
0
−1
Day 28 – Day 0
Day 56 – Day 0
0 h – Day 0
12 h – Day 0
24 h – Day 0
72 h – Day 0

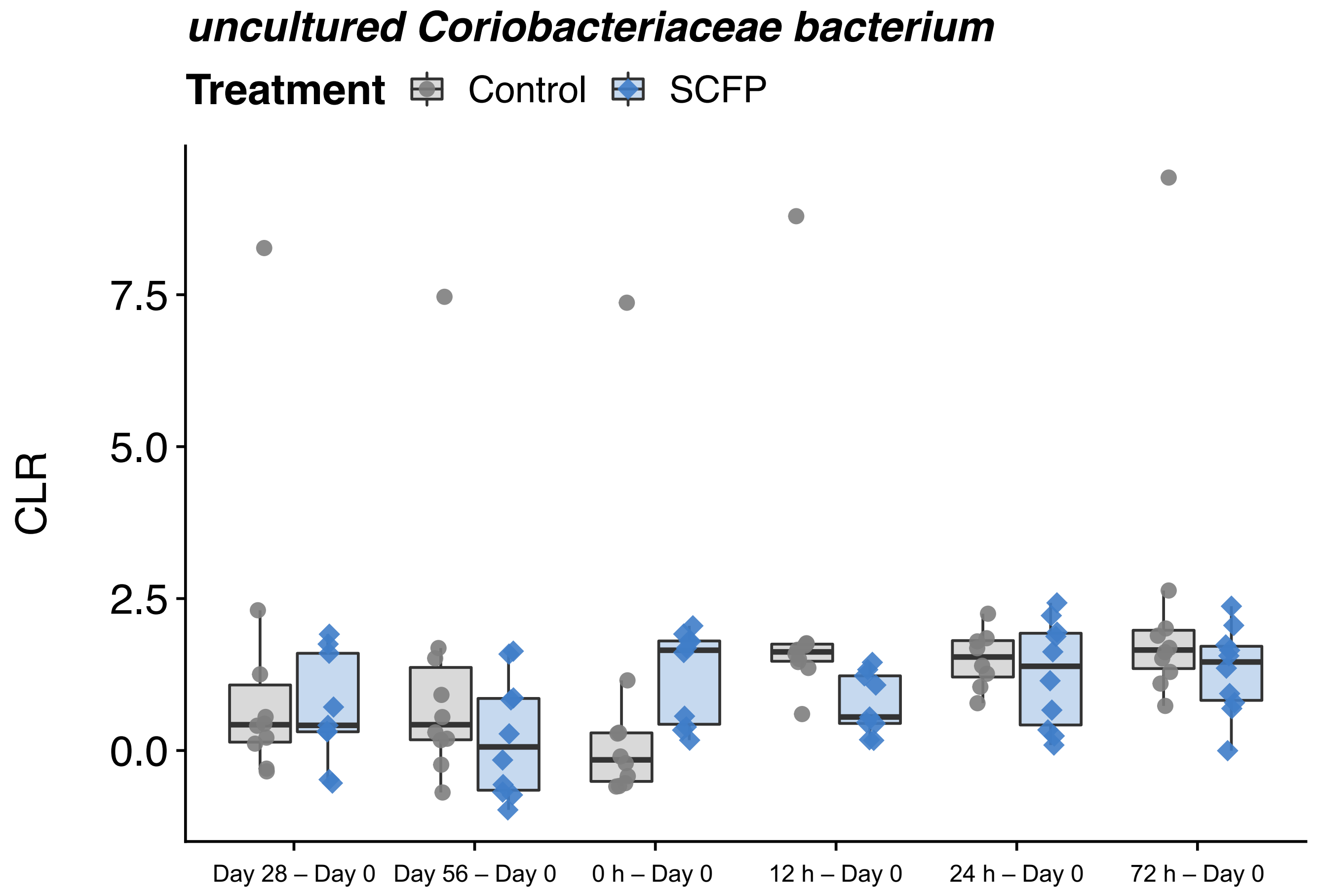
uncultured Coriobacteriaceae bacterium
Treatment
Control
SCFP
CLR
7.5
5.0
2.5
0.0
Day 28 – Day 0
Day 56 – Day 0
0 h – Day 0
12 h – Day 0
24 h – Day 0
72 h – Day 0

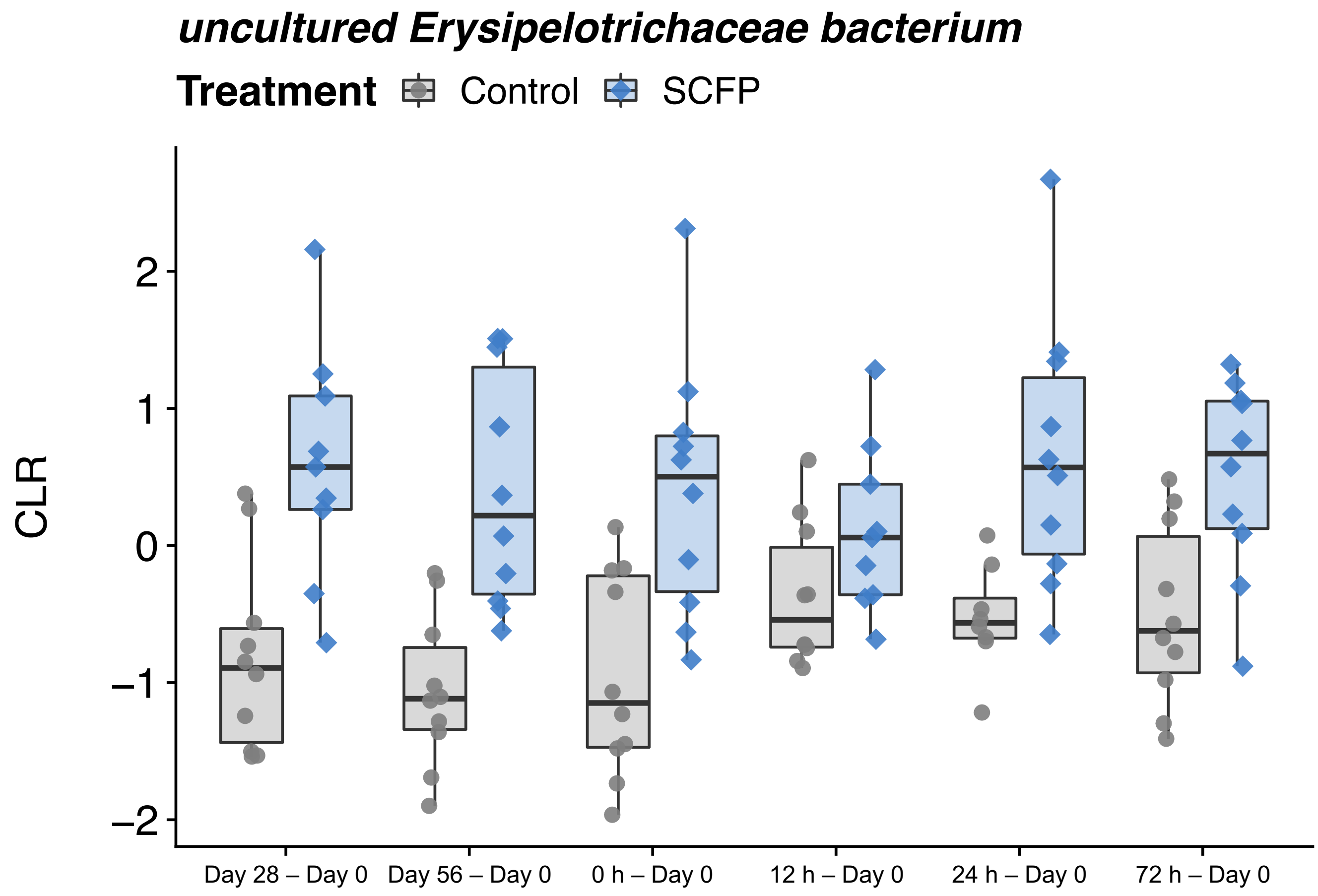
uncultured Erysipelotrichaceae bacterium
Treatment
Control
SCFP
CLR
2
1
0
−1
−2
Day 28 – Day 0
Day 56 – Day 0
0 h – Day 0
12 h – Day 0
24 h – Day 0
72 h – Day 0

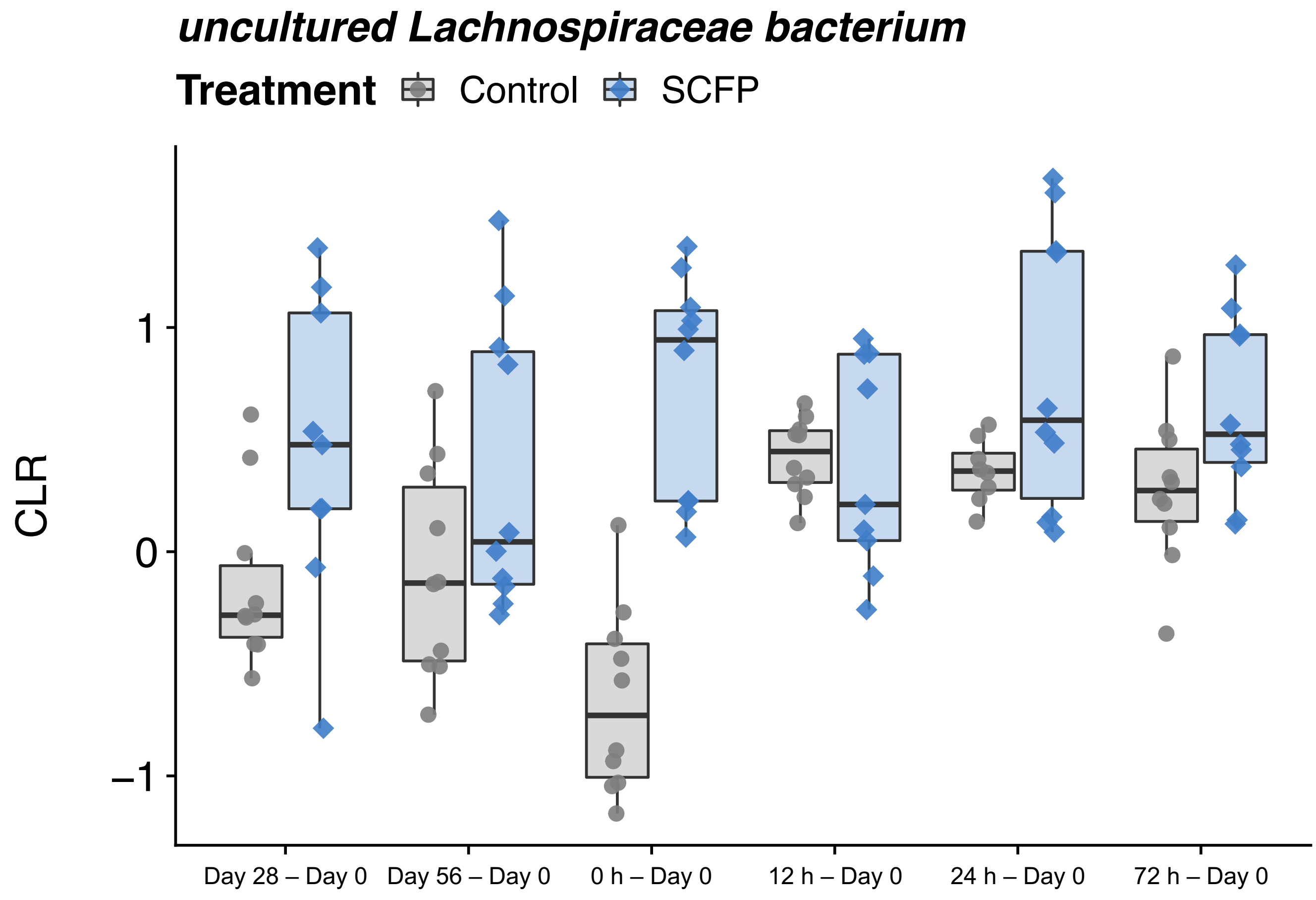
uncultured Lachnospiraceae bacterium
Treatment
Control
SCFP
CLR
1
0
−1
Day 28 – Day 0
Day 56 – Day 0
0 h – Day 0
12 h – Day 0
24 h – Day 0
72 h – Day 0

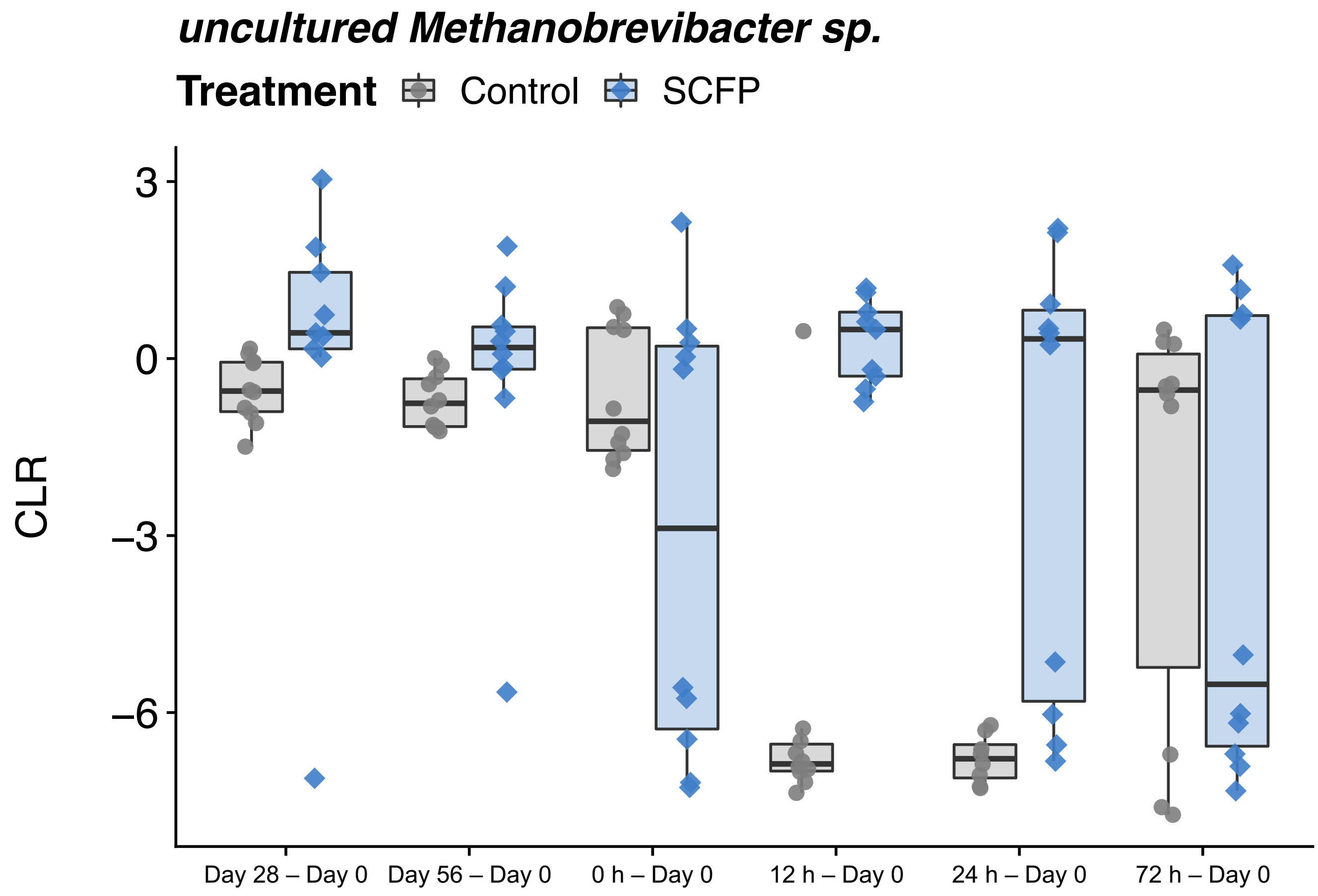

uncultured Methanobrevibacter sp.
Treatment
Control
SCFP
CLR
3
0
−3
−6
Day 28 – Day 0
Day 56 – Day 0
0 h – Day 0
12 h – Day 0
24 h – Day 0
72 h – Day 0

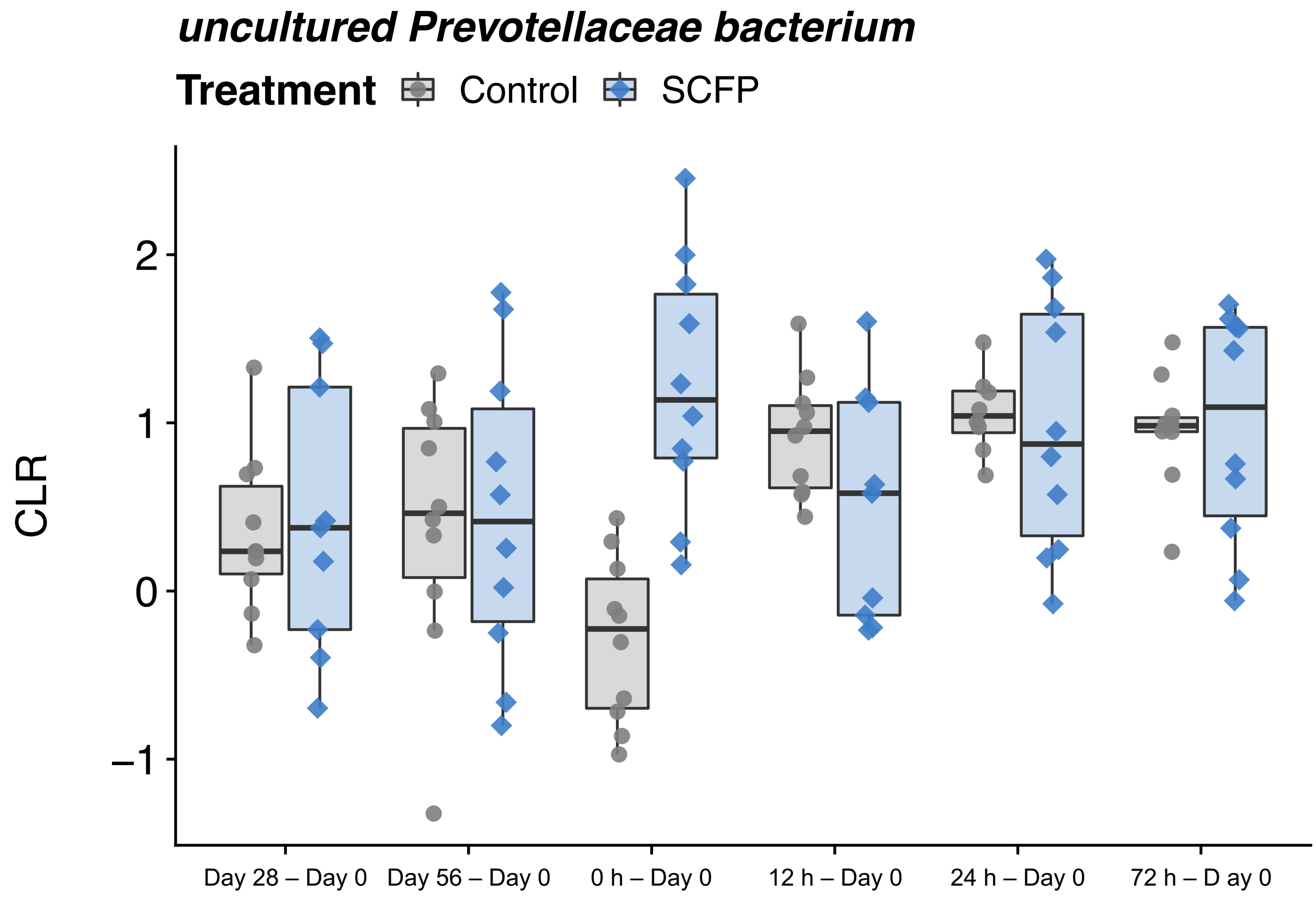

uncultured Prevotellaceae bacterium
Treatment
Control
SCFP
CLR
2
1
0
−1
Day 28 – Day 0
Day 56 – Day 0
0 h – Day 0
12 h – Day 0
24 h – Day 0
72 h – D ay 0

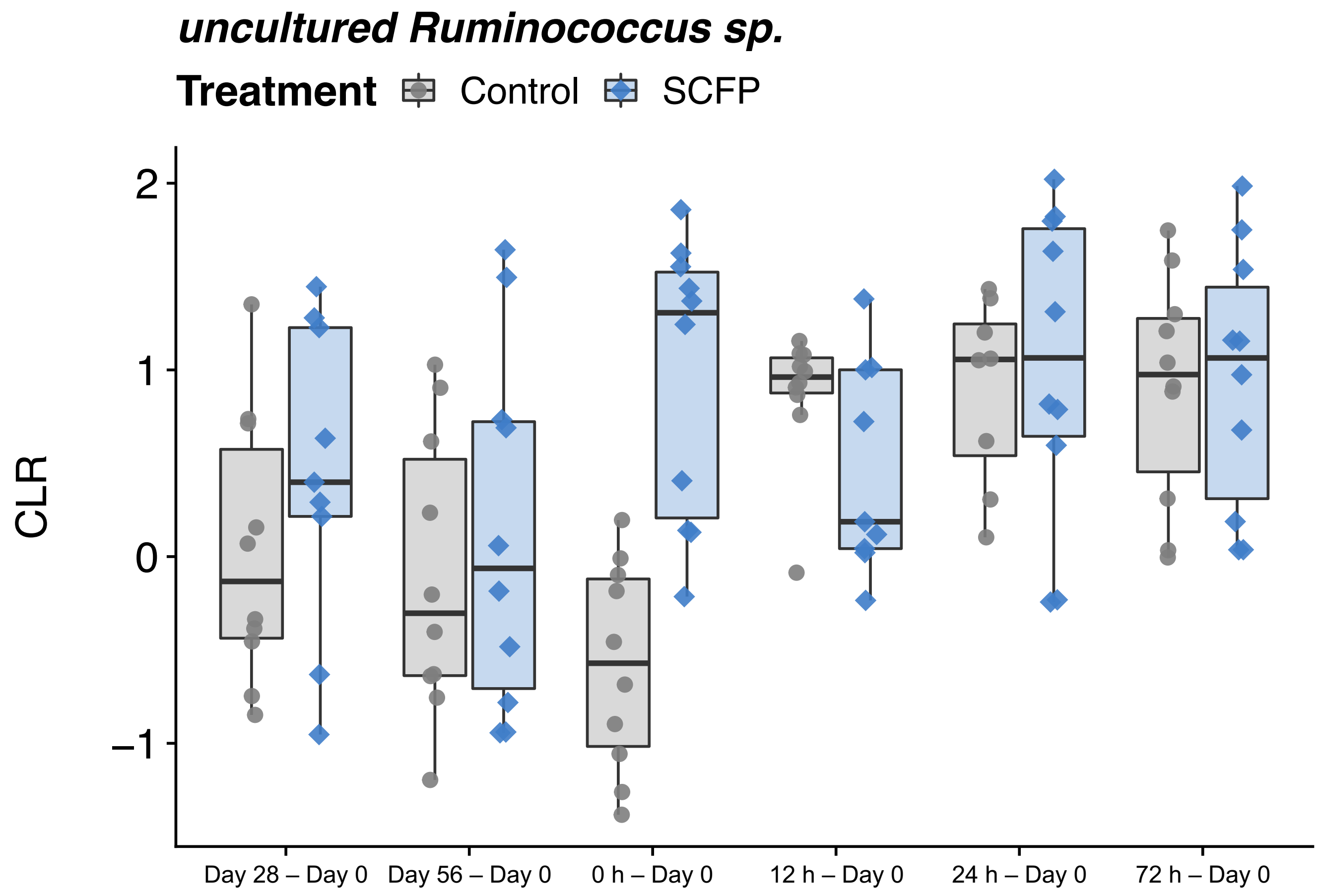

uncultured Ruminococcus sp.
Treatment
Control
SCFP
CLR
2
1
0
−1
Day 28 – Day 0
Day 56 – Day 0
0 h – Day 0
12 h – Day 0
24 h – Day 0
72 h – Day 0
