# Supplementary material for: Saccharomyces cerevisiae fermentation product improves robustness of equine gut microbiome upon stress
Source: Front Vet Sci. 2023 Feb 24;10:1134092. doi: 10.3389/fvets.2023.1134092 (PMC9998945; doi:10.3389/fvets.2023.1134092)
Supplement: Supplementary file 1 [file Data_Sheet_1.zip › Supplementary Tables 1-4.DOCX]

**Supplementary Table S1 – Shannon Diversity**

|  | **Control** | **Mean** | **Median** | **Std** | **SCFP** | **Mean** | **Median** | **Std** | ***P*** ^1^ | ***P* Adj.** ^2^ |
| --- | --- | --- | --- | --- | --- | --- | --- | --- | --- | --- |
| **Day 0** |  | 2.5626 | 2.5599 | 0.0293 |  | 2.5743 | 2.6082 | 0.1077 | 0.6232 | 0.7011 |
| **Day 28** |  | 2.4731 | 2.4899 | 0.1626 |  | 2.4505 | 2.4445 | 0.1678 | 0.2428 | 0.2980 |
| **Day 56** |  | 2.4894 | 2.4824 | 0.0975 |  | 2.4732 | 2.5027 | 0.1172 | 0.9698 | 1.0000 |
| **Stress Event** | |  |  |  |  |  |  |  |  |  |
| **0 h** |  | 2.5698 | 2.5878 | 0.0689 |  | 2.3629 | 2.3689 | 0.0780 | **0.0003** | **0.0015** |
| **12 h** |  | 2.2993 | 2.2951 | 0.0398 |  | 2.4464 | 2.4707 | 0.0835 | **0.0015** | **0.0044** |
| **24 h** |  | 2.2404 | 2.2428 | 0.0945 |  | 2.3334 | 2.3415 | 0.1173 | 0.0831 | 0.1122 |
| **72 h** |  | 2.3519 | 2.3592 | 0.1080 |  | 2.3693 | 2.3313 | 0.1164 | 1.0000 | 1.0000 |

^1^ **Wilcoxon Rank Sum**

^2^ **after Benjamini-Hochberg FDR Correction**

**Supplementary Table S2 – Within-Group Shannon Diversity Comparisons - Control**

| **Control** | **Mean** | **Median** | **Std** |  | **Mean** | **Median** | **Std** | ***P*** ^1^ | ***P* Adj.** ^2^ |
| --- | --- | --- | --- | --- | --- | --- | --- | --- | --- |
| ***Day 0 vs Day 28*** | 2.5626 | 2.5599 | 0.0293 |  | 2.4899 | 0.1626 | 2.4731 | 0.1212 | 0.1559 |
| ***Day 0 vs Day 56*** | 2.5626 | 2.5599 | 0.0293 |  | 2.4824 | 0.0975 | 2.4894 | **0.0257** | 0.0535 |
| ***Day 0 versus 0 h after Stress*** | 2.5626 | 2.5599 | 0.0293 |  | 2.5878 | 0.0689 | 2.5698 | 0.7913 | 0.8546 |
| ***Day 0 versus 12 h after Stress*** | 2.5626 | 2.5599 | 0.0293 |  | 2.2951 | 0.0398 | 2.2993 | **0.0002** | **0.0013** |
| ***Day 0 versus 24 h after Stress*** | 2.5626 | 2.5599 | 0.0293 |  | 2.2428 | 0.0945 | 2.2404 | **0.0000** | **0.0012** |
| ***Day 0 versus 72 h after Stress*** | 2.5626 | 2.5599 | 0.0293 |  | 2.3592 | 0.1080 | 2.3519 | **0.0002** | **0.0013** |
| ***Day 56 versus 0 h after Stress*** | 2.4894 | 2.4824 | 0.0975 |  | 2.5878 | 0.0689 | 2.5698 | 0.0757 | 0.1075 |
| ***Day 56 versus 12 h after Stress*** | 2.4894 | 2.4824 | 0.0975 |  | 2.2951 | 0.0398 | 2.2993 | **0.0002** | **0.0013** |
| ***Day 56 versus 24 h after Stress*** | 2.4894 | 2.4824 | 0.0975 |  | 2.2428 | 0.0945 | 2.2404 | **0.0001** | **0.0012** |
| ***Day 56 versus 72 h after Stress*** | 2.4894 | 2.4824 | 0.0975 |  | 2.3592 | 0.1080 | 2.3519 | **0.0073** | **0.0179** |

^1^ **Wilcoxon Rank Sum**

^2^ **after Benjamini-Hochberg FDR Correction**

**Supplementary Table S3 – Within-Group Shannon Diversity Comparisons - SCFP**

| **SCFP** | **Mean** | **Median** | **Std** |  | **Mean** | **Median** | **Std** | ***P*** ^1^ | ***P* Adj.** ^2^ |
| --- | --- | --- | --- | --- | --- | --- | --- | --- | --- |
| ***Day 0 vs Day 28*** | 2.5743 | 2.6082 | 0.1077 |  | 2.4505 | 2.4445 | 0.1678 | 0.0535 | 0.0902 |
| ***Day 0 vs Day 56*** | 2.5743 | 2.6082 | 0.1077 |  | 2.4732 | 2.5027 | 0.1172 | 0.0757 | 0.1075 |
| ***Day 0 versus 0 h after Stress*** | 2.5743 | 2.6082 | 0.1077 |  | 2.3629 | 2.3689 | 0.0780 | **0.0008** | **0.0026** |
| ***Day 0 versus 12 h after Stress*** | 2.5743 | 2.6082 | 0.1077 |  | 2.4464 | 2.4707 | 0.0835 | 0.0535 | 0.0902 |
| ***Day 0 versus 24 h after Stress*** | 2.5743 | 2.6082 | 0.1077 |  | 2.3334 | 2.3415 | 0.1173 | **0.0008** | **0.0026** |
| ***Day 0 versus 72 h after Stress*** | 2.5743 | 2.6082 | 0.1077 |  | 2.3693 | 2.3313 | 0.1164 | **0.0046** | **0.0124** |
| ***Day 56 versus 0 h after Stress*** | 2.4732 | 2.5027 | 0.1172 |  | 2.3629 | 2.3689 | 0.0780 | 0.0452 | 0.0871 |
| ***Day 56 versus 12 h after Stress*** | 2.4732 | 2.5027 | 0.1172 |  | 2.4464 | 2.4707 | 0.0835 | 0.4967 | 0.5831 |
| ***Day 56 versus 24 h after Stress*** | 2.4732 | 2.5027 | 0.1172 |  | 2.3334 | 2.3415 | 0.1173 | 0.0257 | 0.0535 |
| ***Day 56 versus 72 h after Stress*** | 2.4732 | 2.5027 | 0.1172 |  | 2.3693 | 2.3313 | 0.1164 | 0.0640 | 0.1017 |

^1^ **Wilcoxon Rank Sum**

^2^ **after Benjamini-Hochberg FDR Correction**

**Supplementary Table S4 – Significant species interactions on correlation network analysis**

|  | **Control** | | **SCFP** | |  | **Control** | | **SCFP** | |
| --- | --- | --- | --- | --- | --- | --- | --- | --- | --- |
|  | Positive | Negative | Positive | Negative | **Stress Event** | Positive | Negative | Positive | Negative |
| uncultured Bacteroidales bacterium | 22 | 1 | 16 | 1 |  | 15 | 9 | 12 | 1 |
| uncultured Prevotellaceae bacterium | 22 | 1 | 16 | 1 |  | 16 | 8 | 12 | 1 |
| uncultured bacterium | 22 | 0 | 16 | 1 |  | 16 | 8 | 12 | 2 |
| uncultured Lachnospiraceae bacterium | 22 | 0 | 16 | 1 |  | 16 | 8 | 12 | 1 |
| Ruminococcaceae bacterium | 22 | 0 | 16 | 1 |  | 16 | 8 | 12 | 1 |
| uncultured Bacteroidia bacterium | 22 | 0 | 16 | 1 |  | 15 | 8 | 13 | 1 |
| uncultured Clostridiales bacterium | 22 | 0 | 16 | 1 |  | 16 | 8 | 13 | 1 |
| uncultured Bacteroidetes bacterium | 21 | 0 | 16 | 1 |  | 15 | 9 | 12 | 1 |
| uncultured Ruminococcus sp. | 22 | 0 | 16 | 1 |  | 15 | 7 | 12 | 1 |
| uncultured Erysipelotrichaceae bacterium | 22 | 0 | 16 | 0 |  | 15 | 3 | 12 | 1 |
| uncultured Prevotella sp. | 22 | 1 | 16 | 1 |  | 13 | 1 | 12 | 1 |
| *Phascolarctobacterium succinatutens* | 21 | 1 | 2 | 0 |  | 0 | 1 | 3 | 0 |
| uncultured Spirochaetaceae bacterium | 22 | 0 | 16 | 1 |  | 14 | 0 | 1 | 0 |
| uncultured Coriobacteriaceae bacterium | 22 | 0 | 16 | 1 |  | 16 | 8 | 12 | 1 |
| *Fibrobacter succinogenes* | 22 | 0 | 16 | 1 |  | 15 | 9 | 2 | 0 |
| *Ruminococcus flavefaciens* | 22 | 0 | 16 | 0 |  | 15 | 7 | 12 | 1 |
| *Blautia obeum* | 22 | 0 | 0 | 0 |  | 6 | 13 | 4 | 1 |
| *Butyrivibrio proteoclasticus* | 22 | 1 | 16 | 0 |  | 1 | 0 | 3 | 0 |
| *Pseudobutyrivibrio xylanivorans* | 22 | 1 | 3 | 0 |  | 6 | 12 | 3 | 1 |
| *uncultured Methanobrevibacter sp.* | 20 | 0 | 0 | 0 |  | 5 | 12 | 2 | 0 |
| *Acetivibrio ethanolgignens* | 1 | 0 | 0 | 14 |  | 5 | 14 | 3 | 13 |
| *Pseudobutyrivibrio ruminis* | 1 | 0 | 2 | 0 |  | 6 | 14 | 2 | 0 |
| *Butyrivibrio hungatei* | 22 | 1 | 3 | 0 |  | 8 | 12 | 8 | 0 |
| uncultured proteobacterium | 22 | 0 | 16 | 1 |  | 13 | 2 | 2 | 0 |
| uncultured Butyrivibrio sp. | 0 | 0 | 0 | 0 |  | 9 | 8 | 3 | 0 |
| *Rhizophagus irregularis* | 0 | 8 | 0 | 0 |  | 0 | 8 | 9 | 9 |
| *Butyrivibrio fibrisolvens* | 22 | 1 | 0 | 0 |  | 22 | 1 | 8 | 12 |
| Subtotal | 504 | 16 | 282 | 28 |  | 304 | 210 | 198 | 30 |
| Total interactions | 520 | | 310 | |  | 514 | | 228 | |
| Percentage of Positive interactions | 97% | | 91% | |  | 59% | | 87% | |
